# Hydrodynamic Performance Enhancement of Torpedo-Shaped Underwater Gliders Using Numerical Techniques

**DOI:** 10.12688/f1000research.154040.2

**Published:** 2025-04-23

**Authors:** Sudheendra Prabhu K, Srinivas G

**Affiliations:** 1 Aeronautical & Automobile Engineering, Manipal Institute of Technology (MIT), Manipal Academy of Higher Education (MAHE), Manipal, Udupi Karnataka, 576104, India

**Keywords:** Torpedo; Hydrodynamics; CFD; Underwater glider; Drag.

## Abstract

**Background:**

Underwater gliders are widely used in marine applications for monitoring purposes. These gliders must withstand hydrodynamic forces and maintain its body stability. The underwater environments are highly unpredictable, and small changes in the environment can lead to significant instability in underwater vehicles.

**Methods:**

This study uses different numerical techniques to investigate the hydrodynamic characteristics of a torpedo-shaped glider. A symmetric torpedo-shaped glider model was created and analyzed using a licensed version of ANSYS 20.1 Fluent tool. The behavior of the torpedo glider under various flow conditions was examined such as variation of grid test, change of turbulent models, the variation in the inflow boundary conditions involves varying the velocity from 10.16 m/s to 15.16 m/s in 1m/s increment and from 10.16 m/s to 7.66 m/s in 0.5 m/s, also six different models were analyzed.

**Results:**

Research was also attempted with different turbulent models and the Spalart-Allmara model was producing least validation error of 1.28 % with a primary focus on nose optimization. By varying the nose length, the study aimed to identify the best-suited nose geometry to minimize drag force. The nose lengths were varied to 0.205 m and 0.19m, resulting in validation errors of 2.81% and 1.16%, respectively, the results are clearly explained in the sub sequent sections of this article.

**Conclusion:**

In conclusion, this study has evaluated various modifications and their impact on drag force reduction. The application of Spallart-Allmara model resulted in an improvement of 1.28%. Decrease in velocity lead to a significant reduction in the drag force, with an improvement of 37.3%. The nose optimization also contributed to drag force; a nose length of 0.205m yielded a 3.37% improvement. While a 0.19m nose length resulted in a 1.67% reduction. This study helps researchers in hydrodynamics by optimizing geometry for drag reduction.

## 1. Introduction

**Figure 1.**
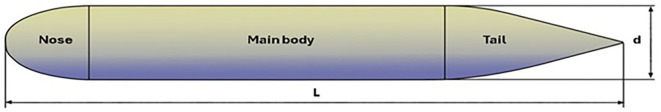
Torpedo sectional view.

The torpedoes were invented in 1860 by Robert Whitehead, an English engineer in Australia, long before there was a theoretical foundation for the scientific development of underwater missiles. The torpedo is the most destructive naval weapon ever deployed, yet its role in modern warfare is largely ignored by naval professionals, a symmetric geometry of torpedo glider is shown in Figure 1. ^1^

The analysis of hydrodynamic parameters plays a very important role in underwater torpedo gliders or any underwater vehicles. The hydrodynamic forces are time-varying and very important to maintain the glider’s stability. ^2^ While designing any torpedo glider, various changes arise. Hydrodynamic coefficient parameters need to be considered when designing a glider. In general, gliders travel at very low velocity, with a maximum speed of up to 0.2 m/s to 0.5 m/s. ^3^ Various parameters affect the stability of the torpedo glider, the parameters include lift, drag, mass of the glider, and velocity. These lift, drag, mass, and thrust forces need to be balanced for the stability of the torpedo glider. There are parameters like glider design, Reynolds number, lift-to-drag ratio, and the angle of attack that keep changing during maneuverability, which can vary up to 8°, 9°, or 10°. ^4– 6^ The torpedo glider might get unstable due to the internal bouncy system and due to tail-wing geometry. ^7^ A small change in stability leads to a pressure difference, even though the design is correct. Therefore, the glider design needs to be designed in such a way that it can stand up to hydrodynamic forces. ^8^ A few torpedo gliders like Omni-max, Depla, flying wing anchor (FWA), and FISH type anchors are dropped from the air into the water which leads to instability due to changes in the density, instability is also due to the actuating force. ^9, 10^

## 2. Literature review

In underwater it is very difficult to predict stability because of the varying angles of attack. To control drag force is very important for torpedo gliders, these problems are also seen in ships and submarines. ^11^ This can be controlled by changing the torpedo geometry and analyzing using numerical simulations. ^12^ The torpedo nose, tail, and wings are very important parts to control the drag, there are various possible methods to minimize the drag.

The possible solution includes considering the axisymmetric geometry, avoiding the tail or wing, compered nose, or changing the wing-tail dimensions ^12, 13^ and these can be analyzed using computational fluid dynamics (CFD) there various methods for analyzing which include the Datcom method, proper orthogonal decomposition (POD) method, particle image velocimetry (PIV), and fluorescent dye method. ^14– 17^ There are also other solutions like changing fin configuration, minimizing the payload, sharp tip angle, high aspect ratio, optimizing the diameter, hemispherical nose, and duct-shaped nose, and super cavitating geometry for the high-speed torpedo. ^18– 21^

Hydrodynamic shape optimization using approximate model can improve the glider range by 7.64%, enhancing underwater glider performance. ^22^ Stability and endurance of underwater vehicles can be maintained using dynamic models specifically designed for stabilizer and wing locations. ^23^ Additionally, the bio-inspired shapes can reduce the overall drag of the underwater vehicle with 3.68% validation accuracy. ^24^ The adoption of sweep wing strategies contributes to trajectory and endurance improvements. ^25^ Integrating a data-driven approach with computational fluid dynamics (CFD) and machine learning helps to optimize the shape of autonomous underwater vehicles under high-speed and high-angle-of-attack conditions. ^26^

The study provides the relationship between nose geometry and drag force. In below given subsequent sections, comparison, validation, and the variation in the drag force due to velocity modifications. Similarly, modifications in the nose parabola geometry are clarified; additionally, various numerical methods in the Ansys fluent are performed for comparison, detailed results, and conclusions are provided.

### 2.1 Objective of the article

This research focuses on the hydrodynamic performance enhancement of torpedo-shaped underwater vehicles using different numerical techniques. The primary objective of this research article is to test the baseline numerical analysis and validate it with the public literature review. Secondly, the flow conditions are tested with different inlet flow boundary conditions and different turbulence models through the grid independence test, finally identifying the best suitable performance parameters for the chosen torpedo. Finally, the model is also tested for nose shape optimization using the trial-and-error method, identifying the most suitable performance parameters for improved hydrodynamic concepts. These objectives provide deeper understanding of the torpedo gliders behavior towards drag force and inform how the design optimization helps in lower drag and better performance in the operating environment.

## 3. Methods

**Figure 2.**
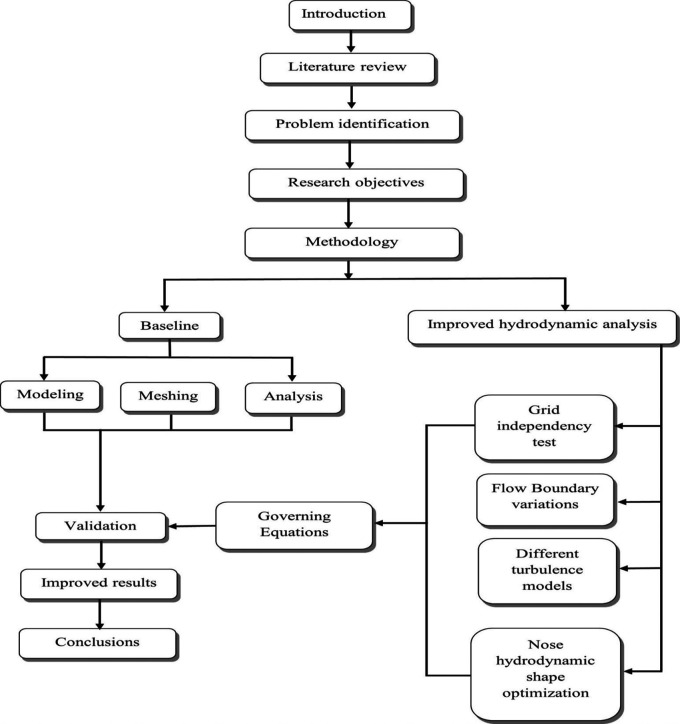
Research methodology flowchart.

As per the diagram Figure 2 below, the complete research article identifies the hydrodynamic problems to fulfill the research objective. Initially baseline was done, and the baseline model for the torpedo glider was prepared using ANSYS designs modeler. Later the torpedo glider model was meshed, the analysis was performed, and baseline results were validated by comparing with the article reference 27. Once the baseline analysis was set, the research followed the next step of the objective, which included the Grid independency test to validate the mesh. Further the analysis continued with six different turbulence models, and the Spalart-Allmara vorticity-based model was chosen for the next step because this model was producing least validation error of 1.28%. The drag force due to changes in boundary conditions was analyzed, from this step, the variation in the drag force due to change in velocity is observed. After performing this step, the main aim of the research article is to carry out the nose hydrodynamic shape optimization.

Here the nose of the torpedo glider was increased and decreased to 5 iterations with a step size of 0.05 m. From this step, the variation in the drag was observed, and the best-suited nose geometry to minimize the drag was chosen; the results are discussed in the subsequent sections.

### 3.1 Baseline analysis

**3.1.1 Modelling of torpedo glider**

**Figure 3.**
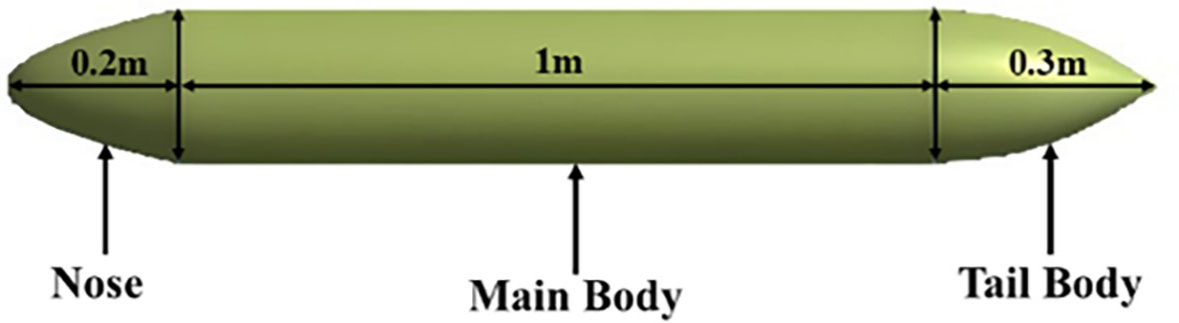
Torpedo glider dimensions.

**Figure 4.**
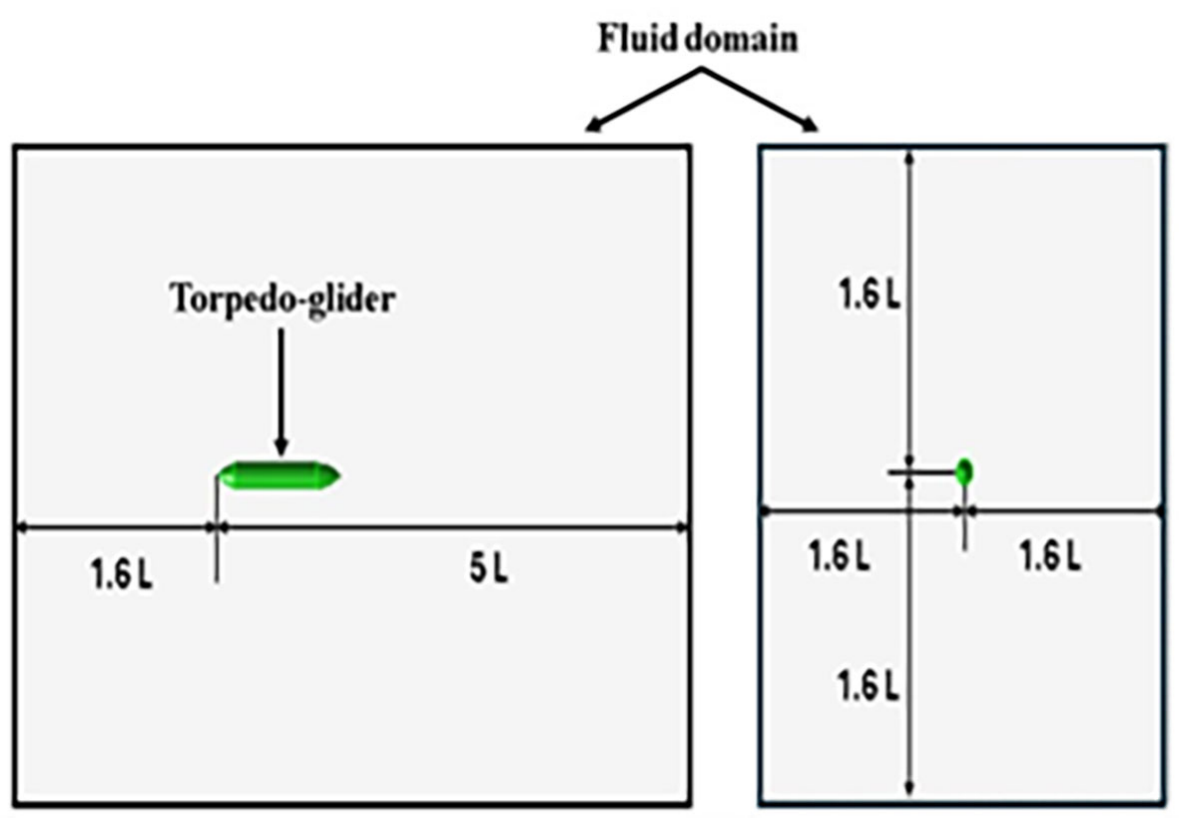
Fluid domain dimensions.

The torpedo glider is modeled using an ANSYS design modular; the total length of the torpedo is 1.5 m; the nose of the torpedo is a parabola with a length of 0.2 m; the aft length is 0.3 m with 20° tail angle; and the middle body is a cylinder with a diameter of 0.2 m. Similarly, the fluid domain is created, and the dimensions of both the torpedo and fluid domains are shown in Figure 3 and Figure 4, the dimensions of the torpedo glider and the fluid domain were taken from the reference 27.

**Figure 5.**
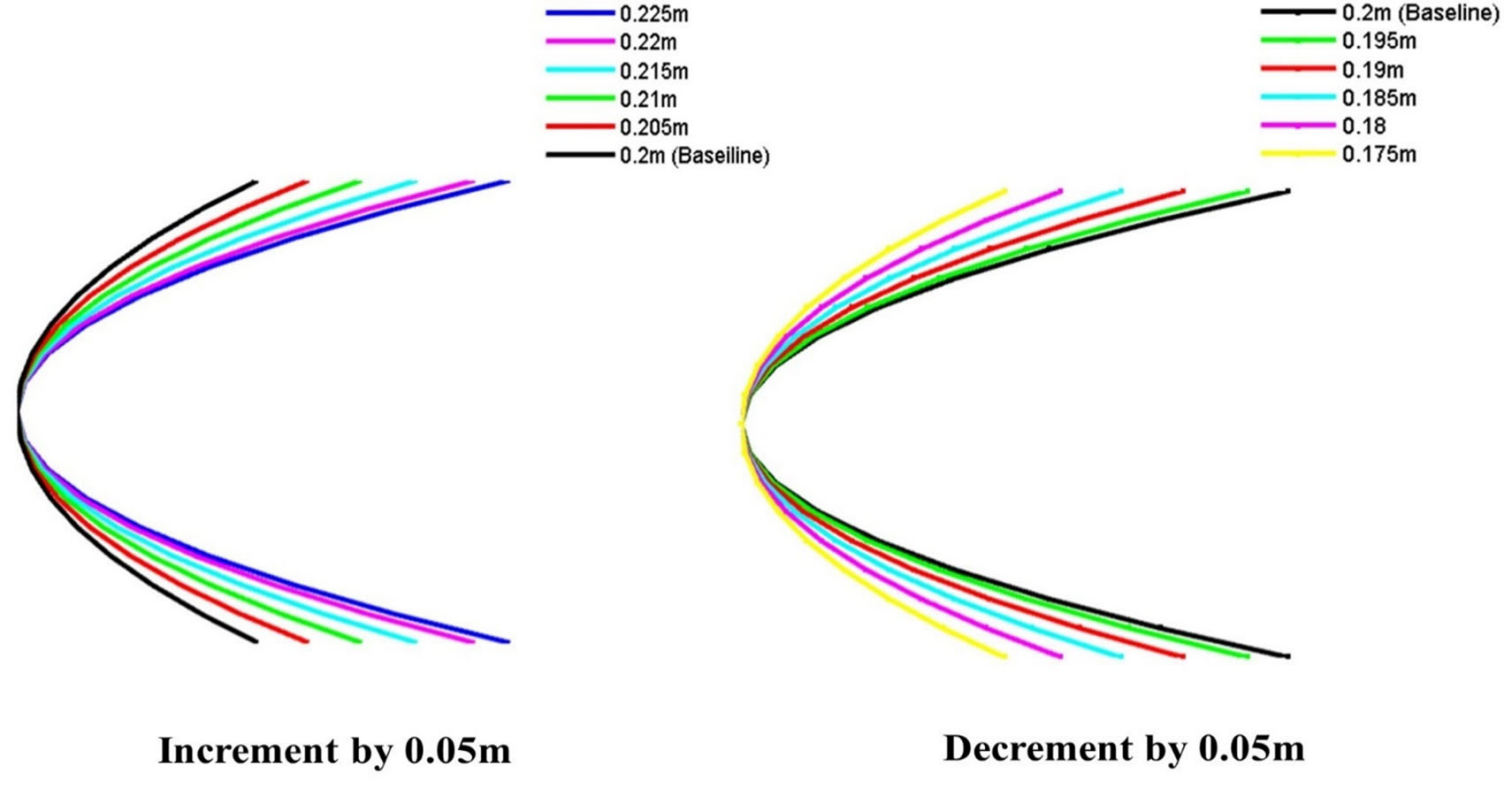
Torpedo nose length optimization.

The nose body shapes of the torpedo glider model have been optimized through various iterations. These shapes have been plotted using MATLAB with appropriate codes, incrementally increasing and decreasing by 0.05 m, as illustrated in Figure 5.

**3.1.2 Meshing of torpedo and fluid domain**

**Figure 6.**
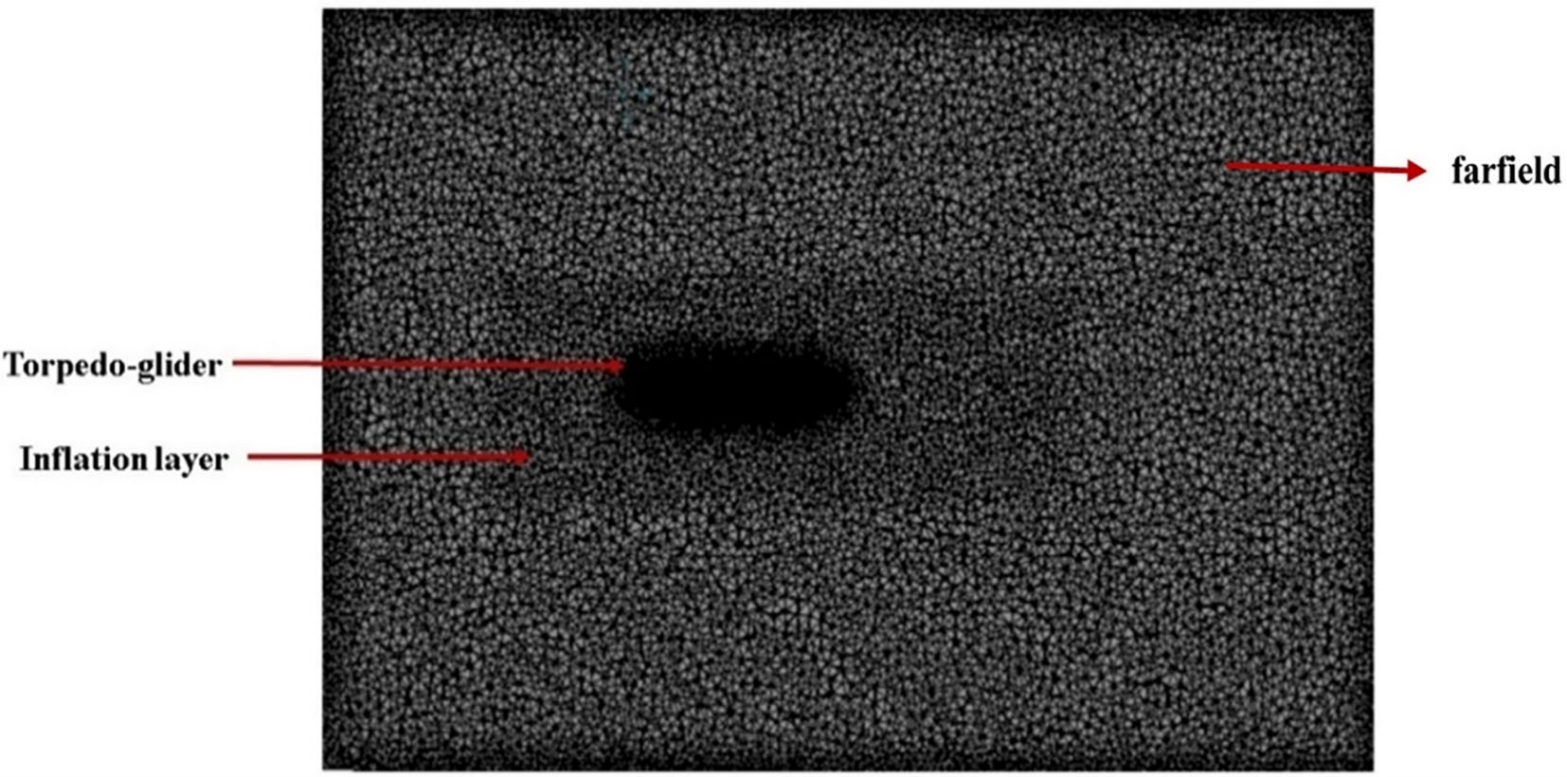
Final mesh with inflation layer in farfield.

**Figure 7.**
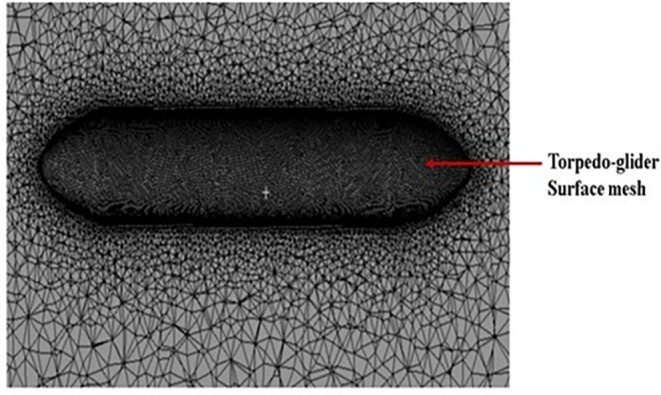
Mesh over the torpedo surface.

After creating torpedo model, the torpedo and fluid domain model was imported for meshing, before meshing inflation body was created as shown in Figure 6, acting like additional fluid domain. A general triangular mesh was chosen, with a y ^+^ of 1 and a boundary layer was created around the torpedo surface. In the Figure 7 below, the mesh over the torpedo and fluid domain can be seen. The mesh size chosen for this model was 48.2 mm, and the number of elements was 4.5 million, after multiple iterations this mesh size was chosen as the baseline for the simulation.

**3.1.3 Numerical modeling**

CFD analysis helps in understanding hydrodynamics on the torpedo gliders or any underwater vehicles, it also helps in understanding the flow behavior of the fluid over the torpedo glider as well as the pressure distribution on the surface of the glider. The speed of the torpedo glider is very small, and the flow is considered as the 3-dimensional steady state and incompressible. The Spallart-Allmaras turbulence model is known as one equation model, describes eddy viscous eddy current flow. This turbulence model is commonly used in hydrodynamic applications including wall bounded conditions, since there is only one equation to solve the process is faster compared to other models.

The governing equation:

$$\frac{\partial v}{\partial x_{j}}=0$$ (1)

The Spallart-Allmaras model, one equation is given by:

$$\frac{\partial \hat{v}}{\partial t}+\frac{\partial \hat{v}}{\partial x_{j}}=C_{b1}\left(1-f_{t2}\right)\hat{S}\hat{v}-\left[C_{w1}f_{w1}-\frac{C_{b1}}{k^{2}}\right]{\left(\frac{\hat{v}}{d}\right)}^{2}+\frac{1}{\sigma }\left[\frac{\delta }{\delta x_{j}}\left(\left(v+\hat{v}\right)\frac{\partial \hat{v}}{\partial x_{j}}\right)\right]+C_{b2}\frac{\partial v}{\partial x_{j}}\frac{\partial v}{\partial x_{i}}$$ (2)

Since the assumption is steady state,

$$\frac{\partial \hat{v}}{\partial t}=0$$ (3)

The turbulent eddy viscosity can be computed from the equation:

$$\mu_{t}=\rho \hat{v}f_{v1}$$ (4)

$$f_{v1}=\frac{x^{3}}{x^{3}+C_{v1}^{3}}$$ (5)

$$x=\frac{\hat{v}}{v}$$

Where *ρ* is the density, v is molecular kinematic viscosity, and μ is molecular dynamic viscosity. *C*_*b*1_, *C*_*b*2_, *k* and *C*_*v*1_ are constants. To estimate the lift force ( *F*_*L*_) and drag force ( *F*_*D*_), the equations (1) and (2) are used.

$$F_{L}=\frac{1}{2}\rho V^{2}AC_{L}$$ (6)

$$F_{D}=\frac{1}{2}\rho V^{2}AC_{D}$$ (7)

Where A is the area on which force is acting. *C*_*L*_ and *C*_*D*_ are the lift and drag coefficients, and V is the free stream velocity of the fluid, All the above equations from (1) to (7) are referred from Ref. 28.

**3.1.4 Boundary conditions on torpedo**

After meshing the model was taken to ANSYS Fluent and the boundary conditions were applied. The vorticity-based Spalart-Allmaras production was chosen for the analysis, with aluminum as the glider material. The inlet was defined as a velocity inlet with a 10% turbulent intensity and a 2m hydraulic diameter. The outlet was specified as a pressure outlet with a backflow turbulent ratio of 10. Both glider and walls were considered as fixed walls with a 0.5 roughness constant. The velocity was 3.046 m/s, and the angle of attack was 9°, since the torpedo was stationary free stream velocity was calculated and given as 10.16 m/s and the fluid parameters, density of 998 kg/m ^3^ and kinematic viscosity of 1.00481×10 ^− 6^ m ^2^/s was set. The torpedo glider was considered as the rigid body, since the speed was too low the far-field was chosen as the wall, and constant density for water was set, the boundary conditions for the Spalart-Allmara vorticity-based model is given in the Table 1. The SIMPLE method was selected as the solution method for the baseline analysis.

**Table 1.** Fluent boundary conditions.

Boundary	U	p	v _t_	v	Boundary type
**Inlet**	Fixed value	Zero gradient	Fixed value	Fixed value	Velocity inlet
**Outlet**	Zero gradient	Fixed value	Zero gradient	Zero gradient	Pressure outlet
**Wall**	Fixed value	Zero gradient	Fixed value	Fixed value	Wall type
**Internal field**	Uniform	Uniform	Uniform	Uniform	Internal fluid

**Figure 8.**
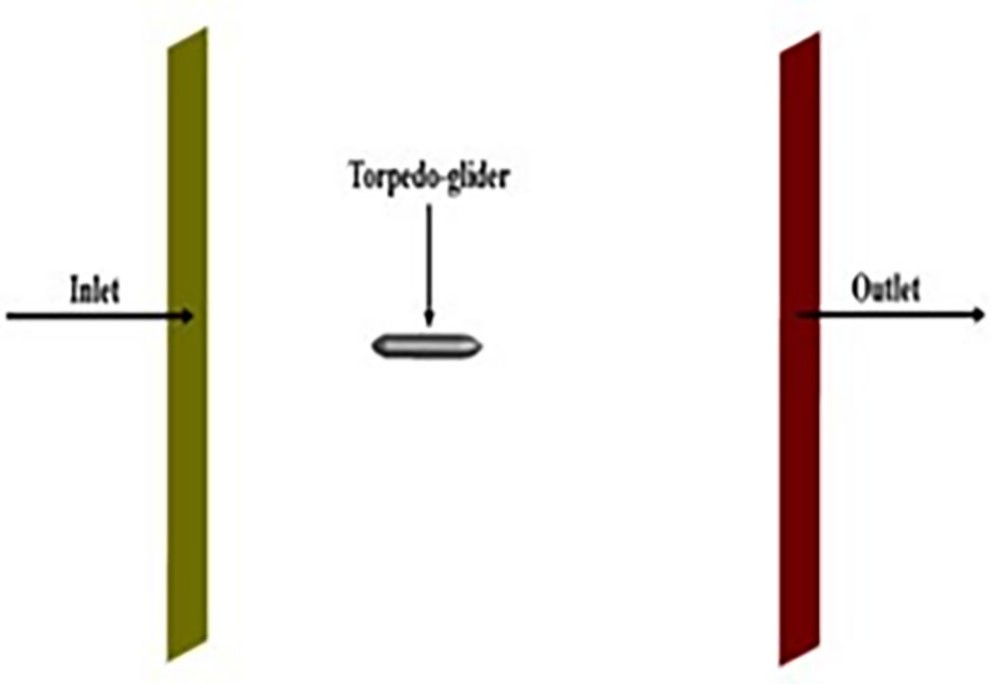
Inlet, outlet and torpedo setup in fluent.

**Figure 9.**
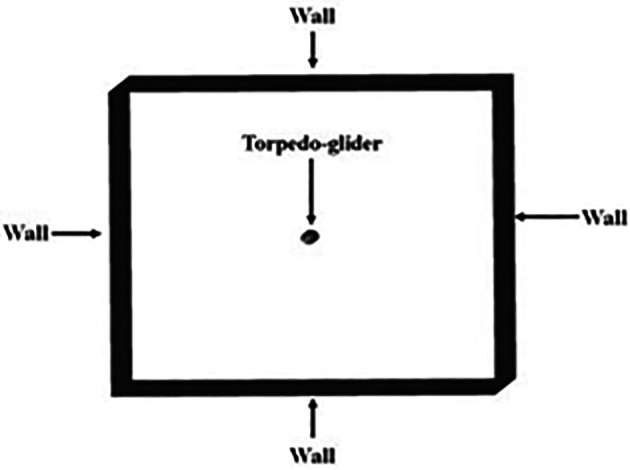
Wall around the torpedo-glider.

**Figure 10.**
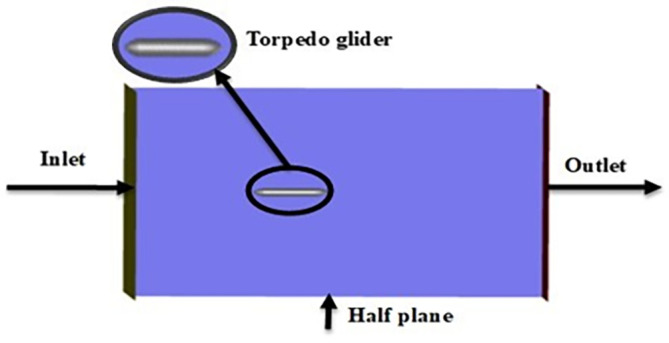
Inlet-outlet boundary with half-plane.

The above boundary conditions were set for the baseline for the simulation, Figure 8, Figure 9, and Figure 10 represents the fluent setup including inlet, outlet, wall, and half-plane. The drag force obtained from the above simulation was compared with the experimental study. ^27^ Later stage the velocity was increased from 10.16 m/s to 15.16 m/s and decreased to 7.66 m/s with each increment and decrement step of 1 m/s. At the final stage of the simulation, the nose geometry was altered, and the same boundary conditions were applied. The nose parabola optimization was performed by incrementing and decrementing the parabola length from 0.2 m to 0.22 m during increment and 0.2 m to 0.175 m during decrement. During decrement with 0.05 m step change, the drag force for all the design changes and boundary condition changes can be seen in the result section.

**3.1.5 Grid independency test**

**Figure 11.**
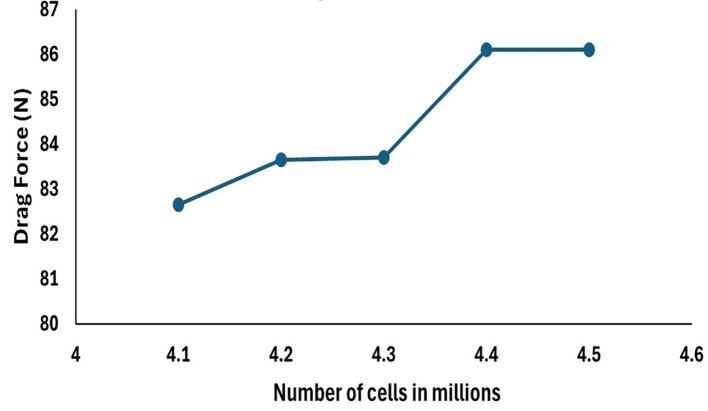
Drag force with respect to number of cells.

To check whether the obtained drag force does not depend on the grid used for the simulation, five different mesh sizes were chosen and resulting in 4.1, 4.2, 4.3, 4.4, and 4.5 million mesh elements in the graph below 4.5 M and 4.4 M are resulting with the same drag force hence the mesh size of 48.2 mm was chosen for the simulation and taken as the base for the analysis of the drag force, the drag force with respect to change in the number cells is observed in Figure 11.

## 4. Results and Discussions

Results were analyzed regarding drag force and the change in the drag force concerning change in the boundary conditions, change in the velocity, and nose optimization for the same (0.2mx1.5m) are discussed. In the below subsection, each step is carried out for analyzing the reports starting from the baseline, method sections, grid independence test, variation of flow velocity, and nose optimization are discussed in detail.

### 4.1 Baseline analysis

The baseline boundary conditions are discussed in the methodology section, the velocity of the glider was set to 3.046 and K-omega SST model was applied due vey slower velocity, torpedo glider was assumed to be steady, and the angle of attack was set to 9°. Since the torpedo was considered as wall and free stream velocity of 10.16 m/s from the inlet was set, different mesh sizes were applied for the same SST model. For the mesh size of 48 mm and 48.2 mm the drag force of 86.11 N can be seen in the Table 2, obtained drag forces are compared with the experimental study. ^27^

**Table 2.** Grid independency test results and error evaluation.

Element size (mm)	Number of elements (in millions)	Drag force (N)	Reference drag force (N) ^27^	Error (%)
50	4.1	82.65	87.4	5.43
49.5	4.2	83.65	87.4	4.29
49	4.3	83.7	87.4	4.23
48	4.4	86.11	87.4	1.47
48.2	4.5	86.11	87.4	1.47

The baseline mesh size was set to 48.2 mm, additional 6 different models for the same torpedo glider geometry was tested and from Table 2, the Spalart-Allmara vorticity-based model results in accurate drag force of 86.28 N and this model was chosen for the next analysis seen in Table 3.

**Table 3.** Drag forces at different viscous models.

Model	Drag force (N)	Number of elements (in millions)
K-elipson Standard model	94.18	4.5
K-omega SST model	86.11	4.5
Spalart-Allmara vorticity-based model	86.28	4.5
Transition SST model	80.24	4.5
Transition K-Kl model	121.46	4.5
Reynolds stress model	73.01	4.5

**Figure 12.**
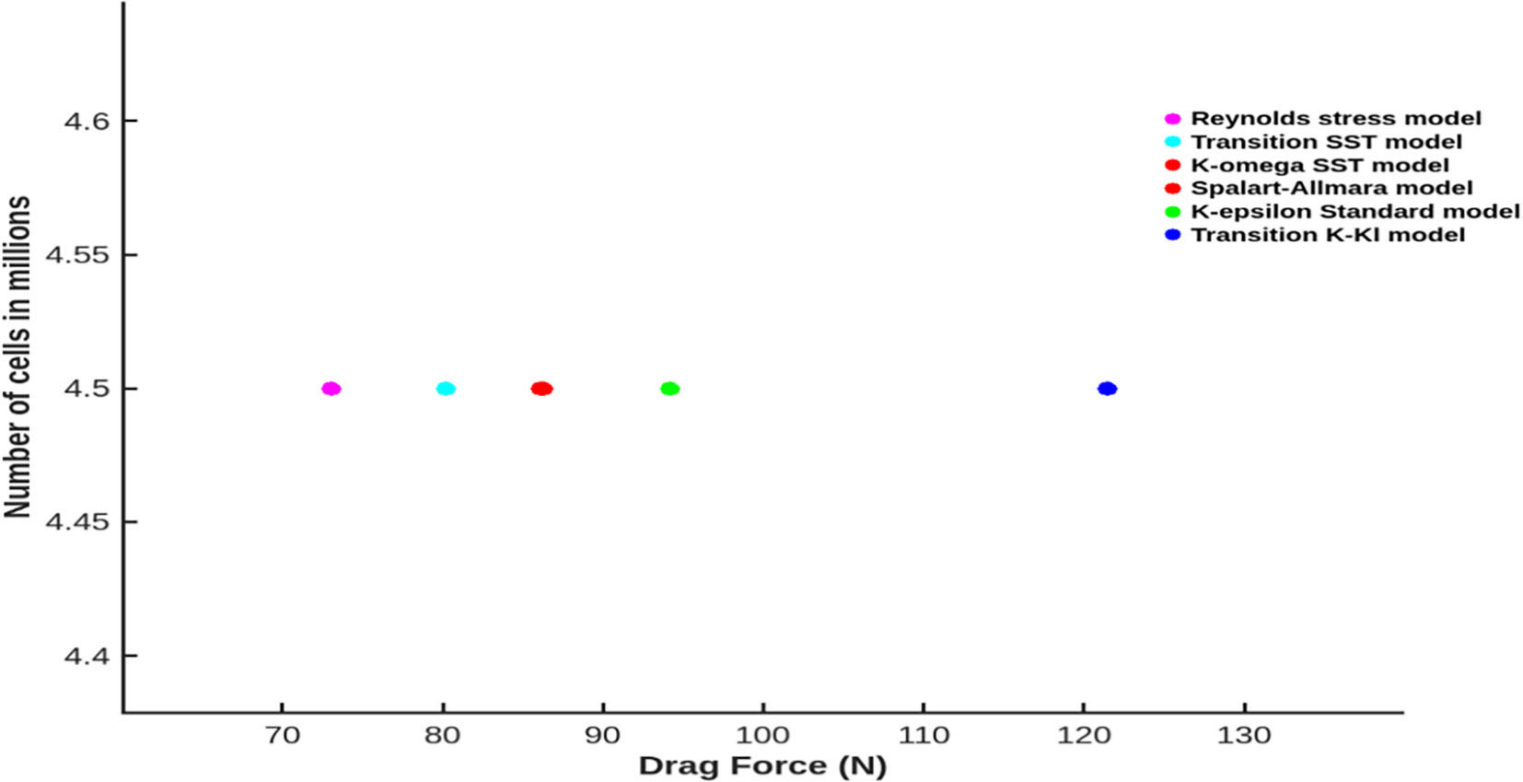
Number of cells vs drag force with 6 different viscous models.

From the Figure 12 it is observed that the K-kl model results in higher drag force compared to other 5 different models, the k-omega SST model and Sparlart-Allmara Model results in the drag force of 86.11 N and 86.28 N but the aim is to minimize the drag. Therefore, the Sparlart-Allmara Model was chosen for further simulation since the error is less compared to other viscous models.

**Figure 13.**
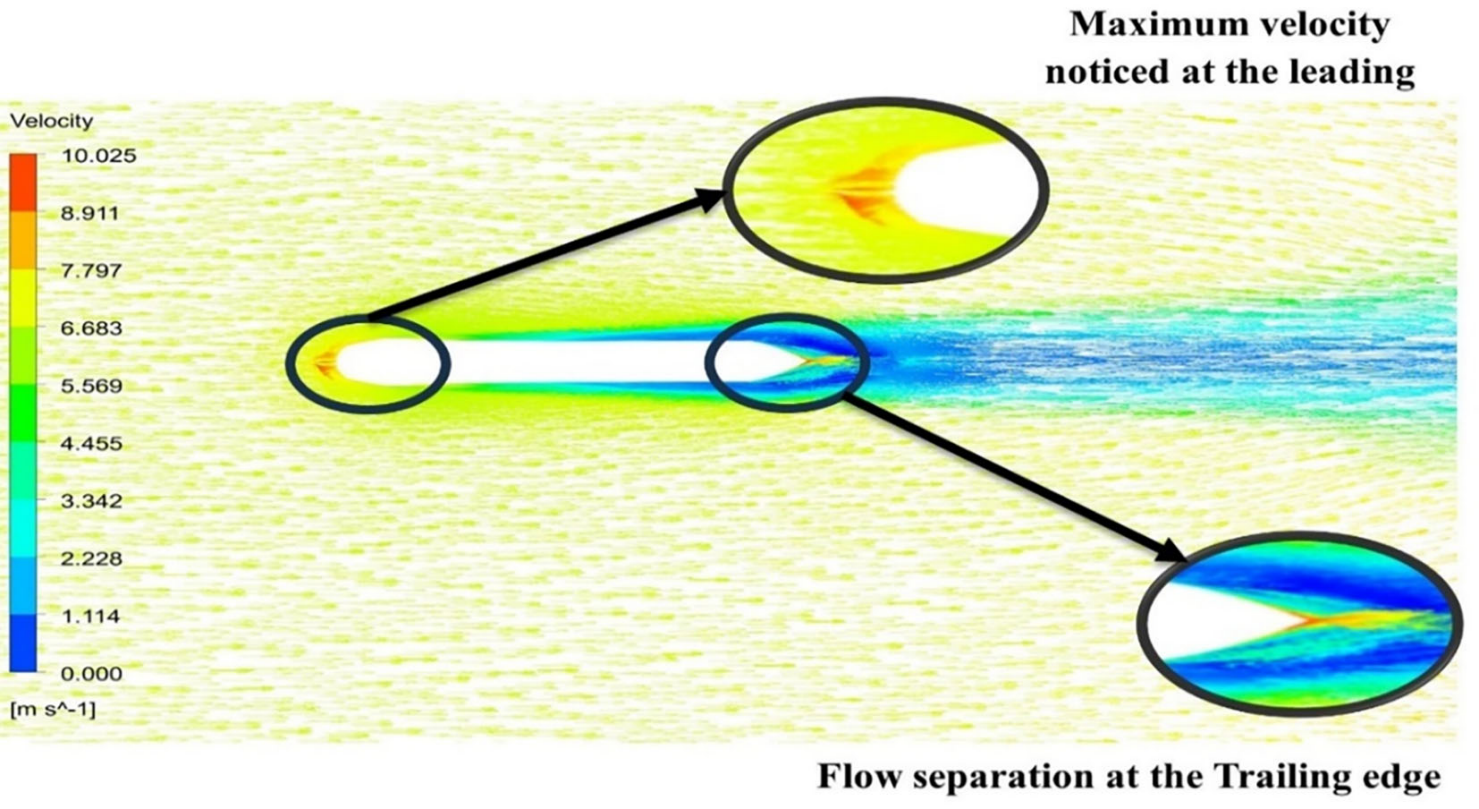
K-omega SST model velocity contour.

**Figure 14.**
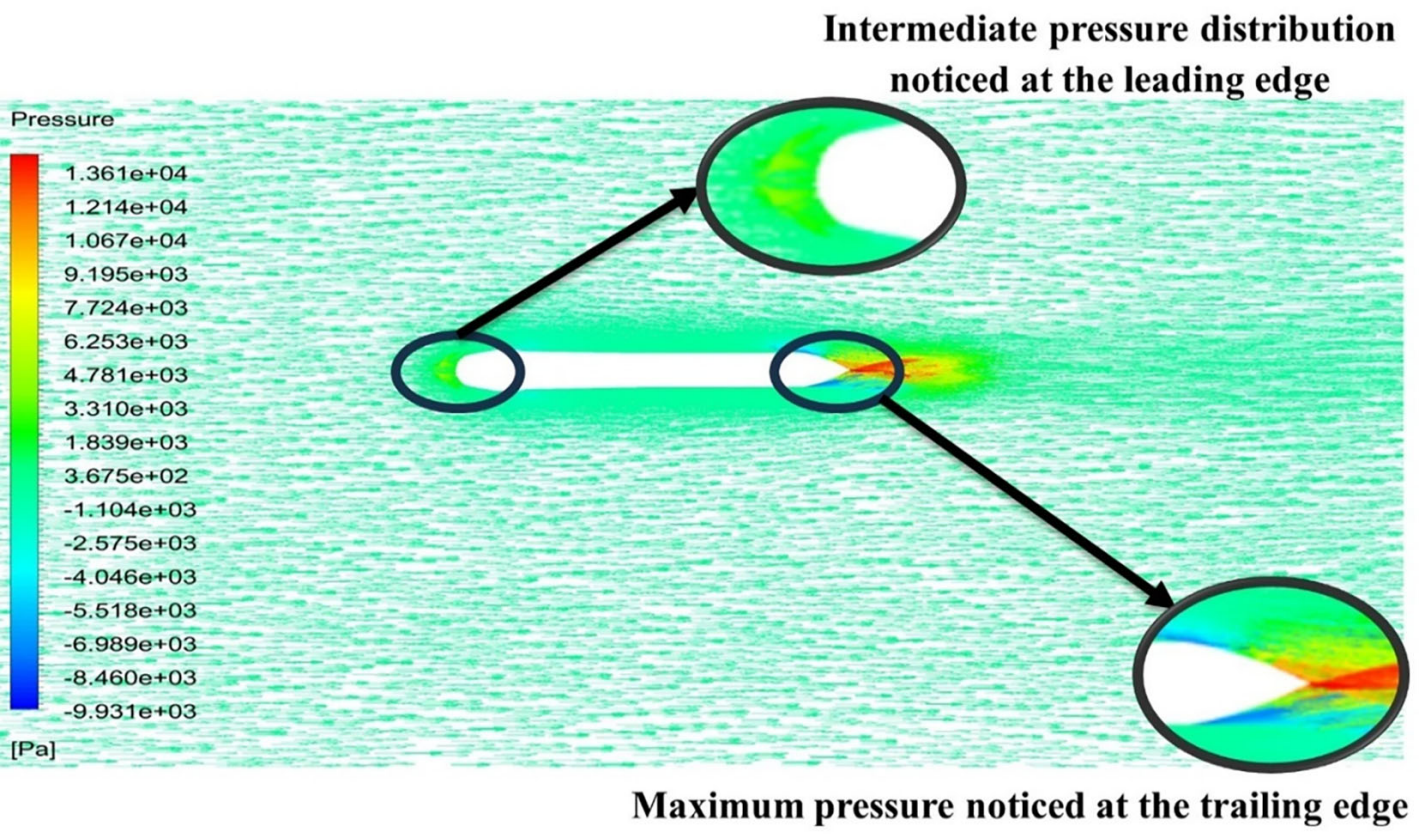
K-omega SST model pressure contour.

From Figure 13 the velocity of 8.911 m/s is observed that, at the leading edge and flow separation is observed at the trailing edge. Similarly, from Figure 14, an intermediate pressure between 4.78 kPa to 10.67 kPa is observed. The figure also shows that the trailing edge tip has a maximum pressure of 13.61 kPa, both contour and velocity contours can be observed in Figure 13 and Figure 14, utilizing the K-omega SST model.

**Figure 15.**
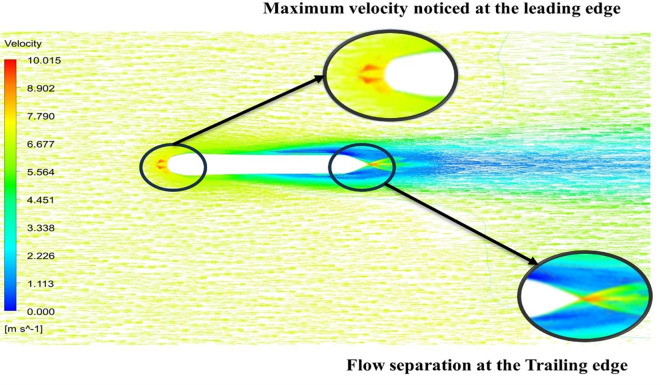
Spalart-Allmara vorticity-based model velocity contour.

**Figure 16.**
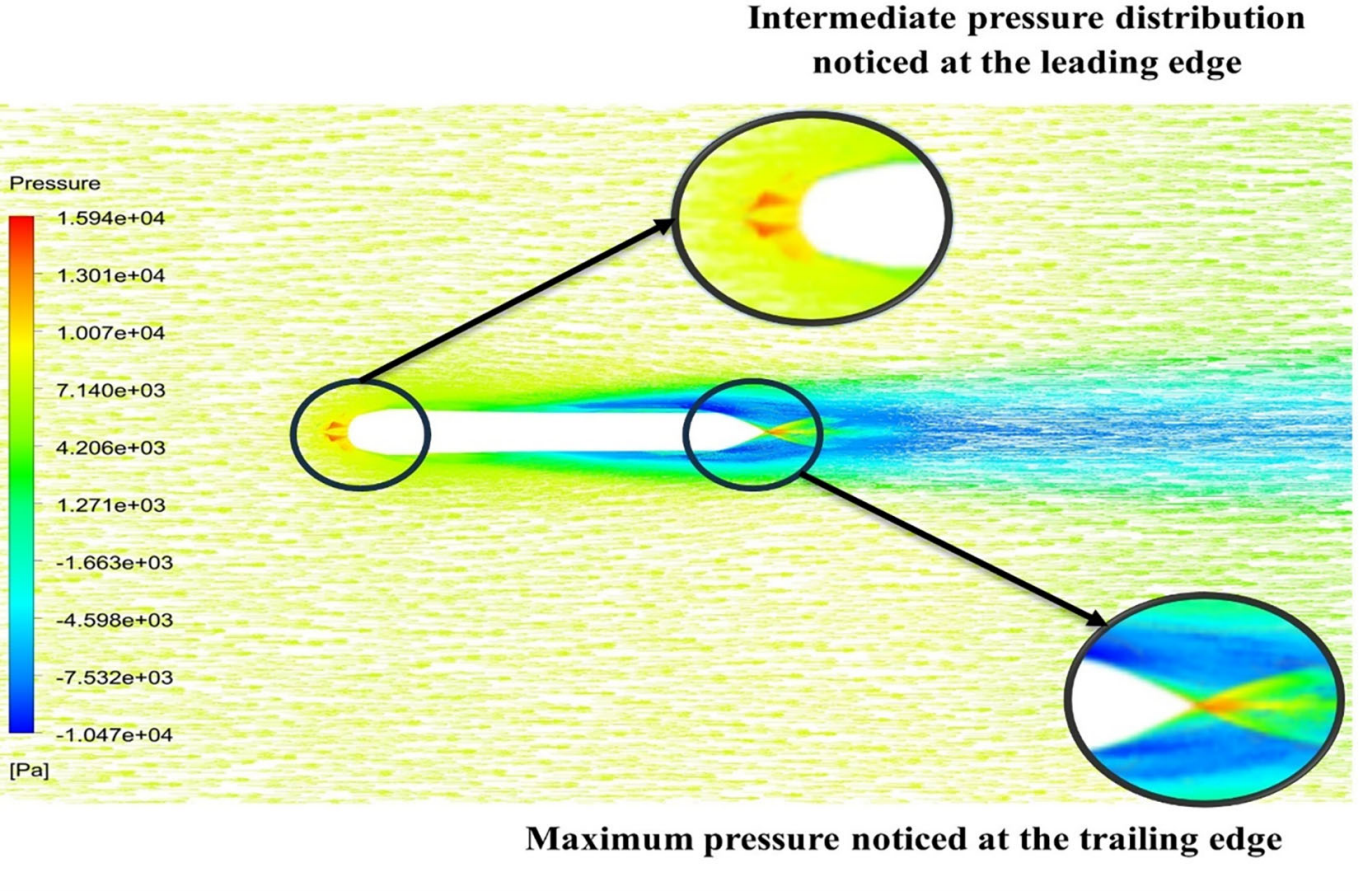
Spalart-Allmara vorticity-based model pressure contour.

From Figure 15 the velocity of 8.902 m/s is observed at the torpedo leading edge and flow separation at the trailing edge. Similarly, from Figure 16, an intermediate pressure between 1.27 kPa to 10.07 kPa is observed, and the figure also shows that the trailing edge tip has a maximum pressure of 15.94 kPa. The pressure and velocity contours can be observed in Figure 15 and Figure 16 utilizing the Spallart-Allmara vorticity-based model.

### 4.2 Variation of flow velocity over the torpedo

In further validation the velocities are varied from 10.16 m/s and the freestream velocity was incremented and decremented to each of 5 iterations as shown in the Table 4. The velocities are varied to understand the variation the drag force, from the analysis it can be observed that higher the velocity more will be the drag and the angle of attack was set to 9°, the angle of attack also plays very important role in stability as the velocity and angle of attack increases the drag will increases. From the table it is observed that, at 15.16 m/s free stream velocity the drag will be 230.9 N, at 7.66 m/s free stream velocity the drag force will be 54.78 N.

**Table 4.** Values of drag force due to change in the velocities.

Increment		Decrement	
Velocity (m/s)	Drag force (N)	Velocity (m/s)	Drag force (N)
11.16	100.99	9.66	79.33
12.16	111.64	9.16	72.42
13.16	135.96	8.66	65.81
14.16	127.9	8.16	60.38
15.16	230.9	7.66	54.78

**Figure 17.**
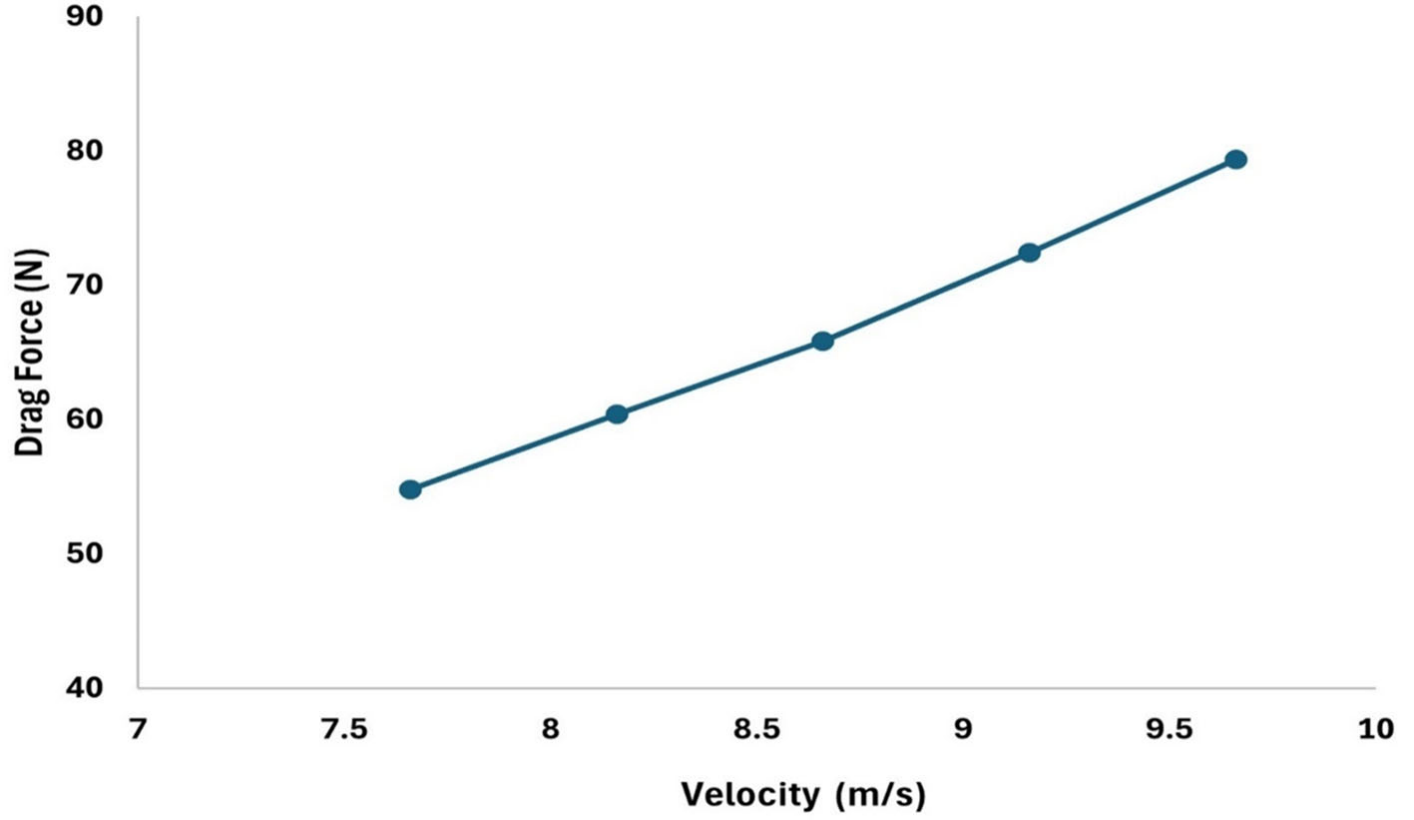
Torpedo-glider velocity decrement at 0.5 m/s.

**Figure 18.**
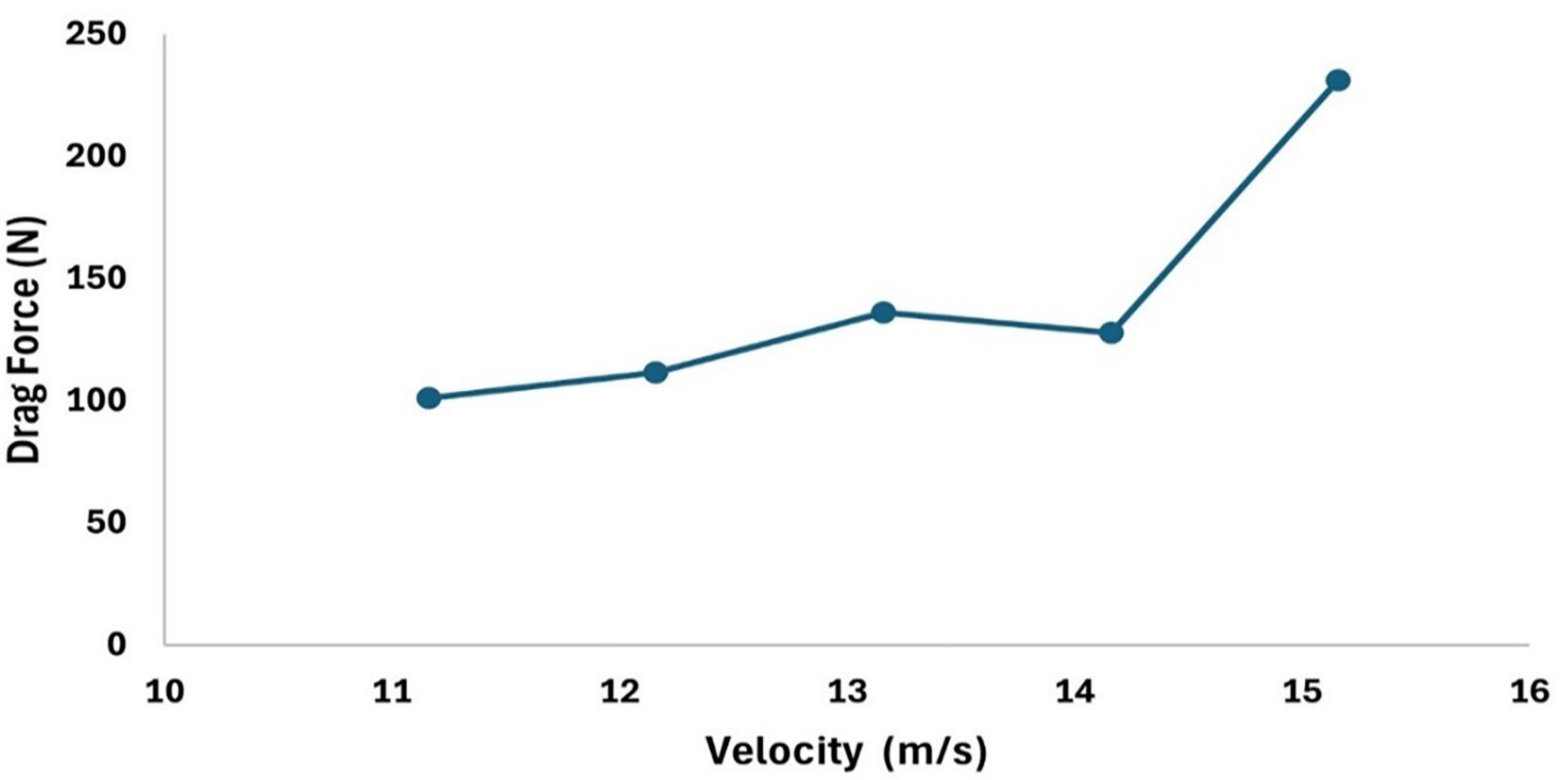
Torpedo-glider velocity increment at 1 m/s.

Figure 18 represents the variation in the drag concerning the increment in the velocity by 1m/s and observed that the drag forces increases as the velocity increases, similarly, Figure 17 represents the variation in the drag force due to a decrement in the velocity by 0.5 m/s, from the graph it is observed that the drag force decreases as the velocity decreases.

**Figure 19.**
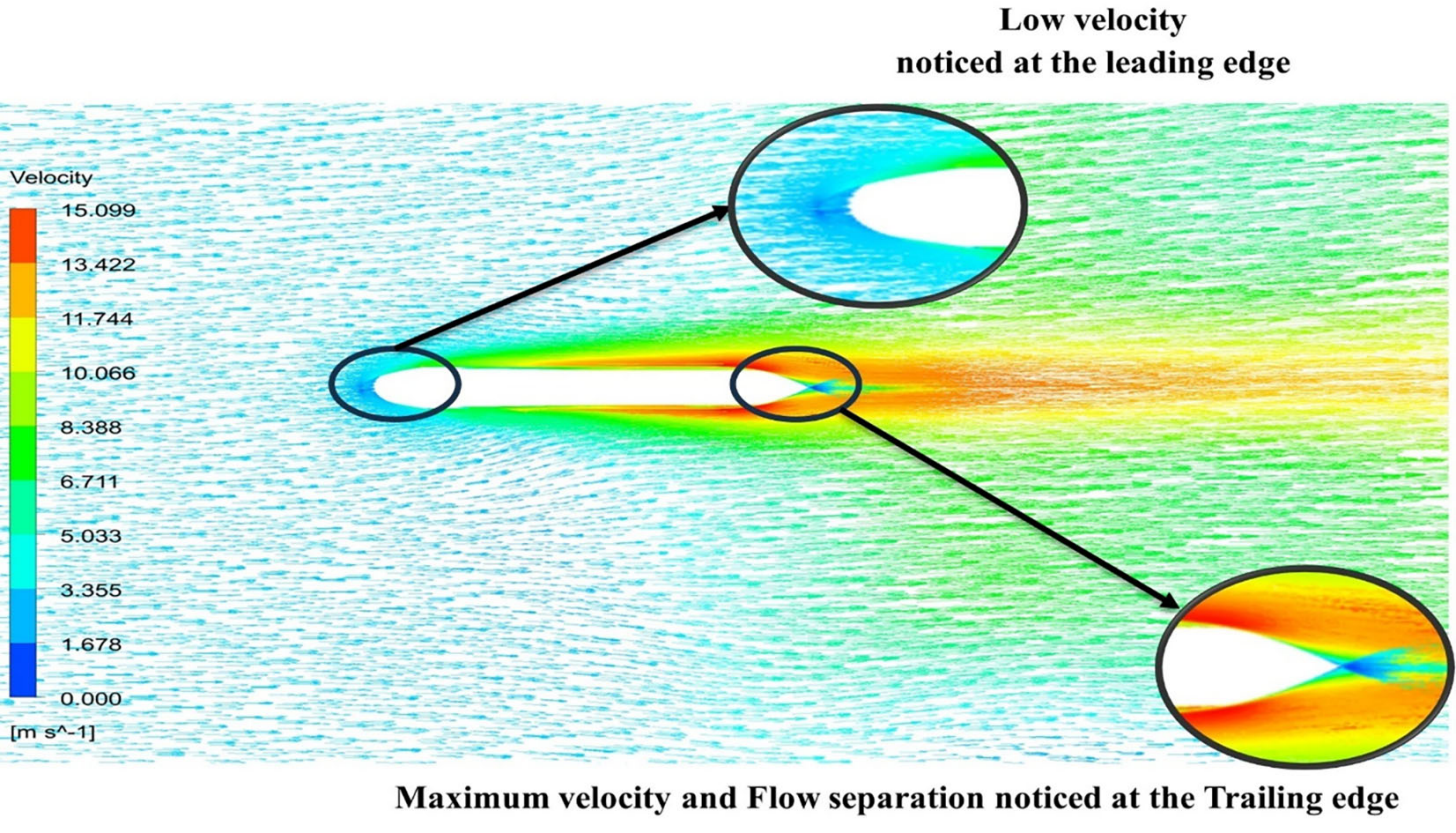
Velocity contour at 15.16 m/s.

Figure 18 and Figure 19 represents the velocity and pressure contours at velocity 15.16 m/s, also it is observed that as the velocity increases the pressure on the torpedo glider also increases, this leads to uniform distribution of the fluid streamlines and the change in pressure is also due the angle of attack. As the angle of attack changes with a velocity of the fluid, flow distribution over the torpedo glider changes and as the result there is a pressure difference at the upper and lower part of the geometry. From Figure 18 and Figure 19 it is observed that shape of tails helps in pushing water out more gradually and less abruptly this helps in minimizing the drag. But the sharp nose is not preferred for low-speed gliders.

**Figure 20.**
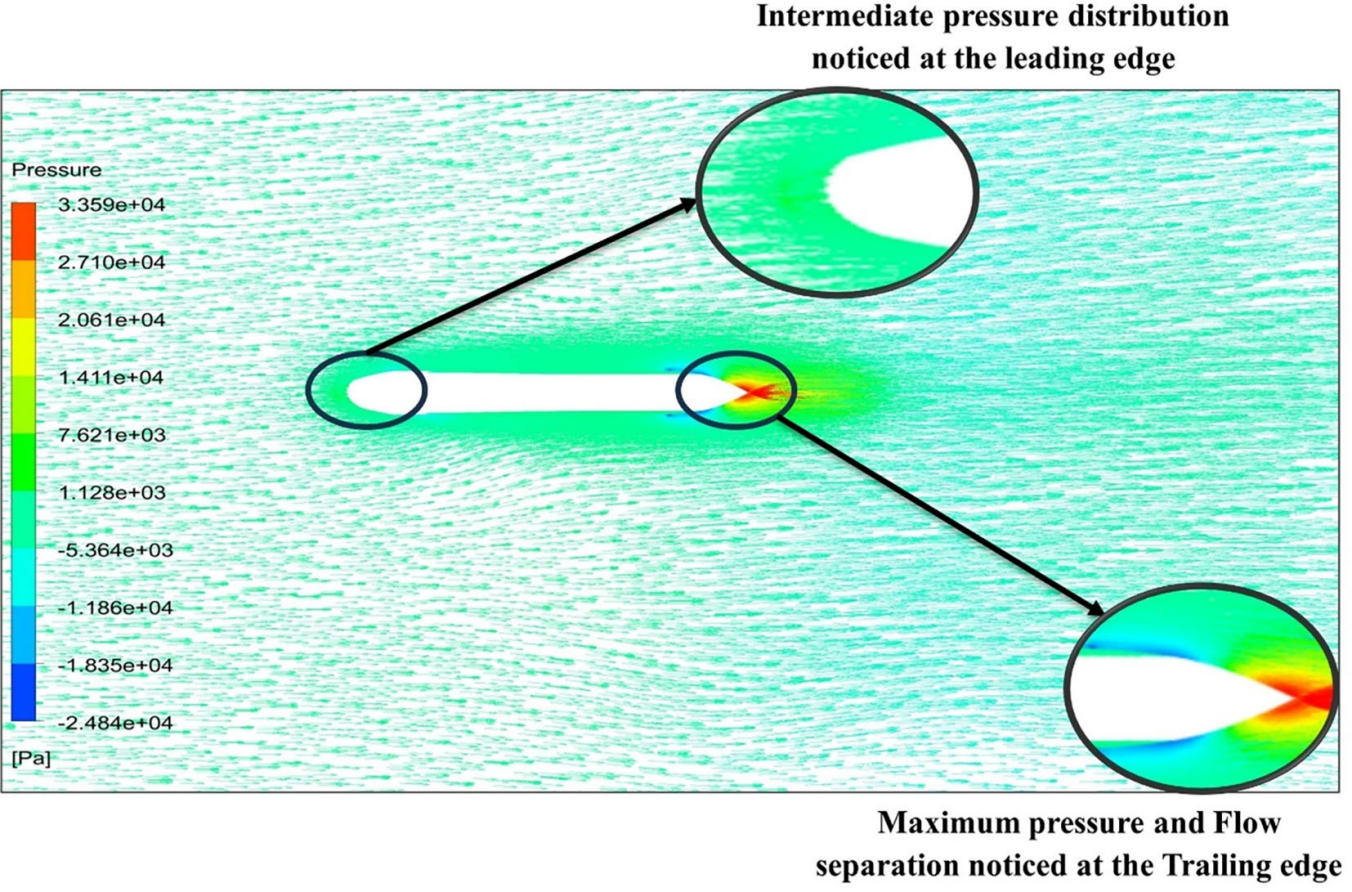
Pressure contour at 15.16 m/s.

**Figure 21.**
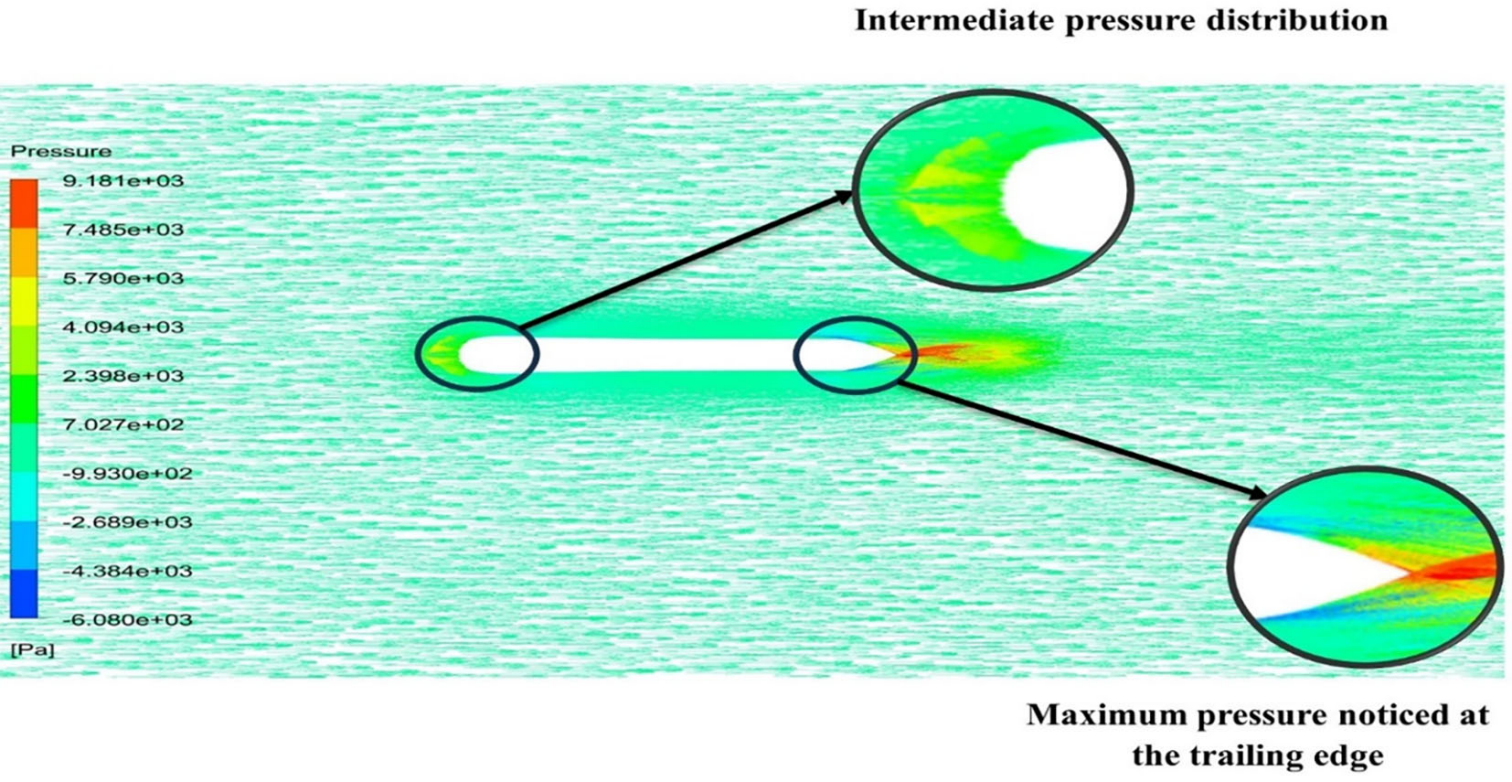
Pressure contour at 7.66 m/s.

**Figure 22.**
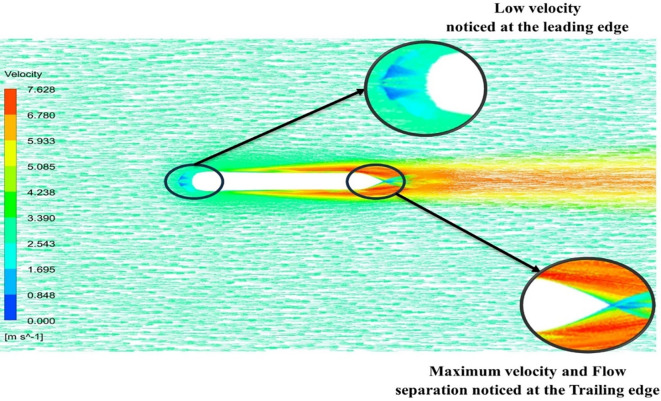
Velocity contour at 7.66 m/s.

From Figure 19 at velocity of 3.355 m/s it is observed that at the leading-edge surface, maximum velocity of 15.099 m/s, and flow separation at the trailing edge. Similarly, from Figure 20, an intermediate pressure between 1.128 KPa to 20.6 KPa is observed, and the figure also shows that the trailing edge tip has a maximum pressure of 33.59 Kpa. Both pressure and velocity contours can be observed from Figure 19 and Figure 20, utilizing the Spallart-Allmara vorticity-based model. From Figure 22 the velocity of 2.543 m/s is observed at the leading-edge surface, maximum velocity of 7.628 m/s, and flow separation at the trailing edge. Similarly, from Figure 21, an intermediate pressure between 5.79 KPa to 7.027 KPa is observed, and the figure also shows that the trailing edge tip has a maximum pressure of 9.818 KPa, both pressure and velocity contours can be observed from Figure 21 and Figure 22, utilizing the Spallart-Allmara vorticity based model.

Figure 20 and Figure 21 shows the velocity and pressure contour at 7.66 m/s free stream velocity, here the drag force is found to be 54.78 N which is much less compared to 230.9 N drag due to 15.16 m/s free stream velocity. This part of the simulation is just to understand the change in drag due to change in the flow parameters like free stream velocity and angle of attack. Here it can be observed that the drag can be reduced by decreasing the velocity, but the reducing velocity does not help in the torpedo gliders because torpedoes are mainly used for defence applications and they have to maintain a constant speed, as a result, the only way to minimize the drag is by altering the geometry, in the next subsection it is clearly how the drag can be reduced by changing geometry.

### 4.3 Torpedo nose optimization

In the defence application, speed is a very important aspect, and the torpedo glider should have minimum speed. Therefore, the speed cannot be reduced to minimize the drag, another alternative method is to modify the geometry. Here nose part is modified because the torpedo travels at a very low velocity compared to missiles hence the tail shape tail part is well suited for this simulation. As the watertight volume changes the drag force will change. This study aims to understand the change in the drag force when the nose is optimized. In this simulation the parabolic nose is considered because the torpedo travels at a very low speed as a result there is no shock wave on the nose surface, since the speed is less parabolic shape can be used, consideration of the parabolic nose is an advantage over the drag but the light of the nose is also very important because as the length changes the total mass and volume also changes which will affect the drag, hence the nose light should be optimum to minimize the drag. in this simulation, the parabolic nose lengths are incremented and decreased as shown in this table and the lengths are changed to 5 iterations with a change in step length of 0.05 m as shown in Figure 5.

**Figure 23.**
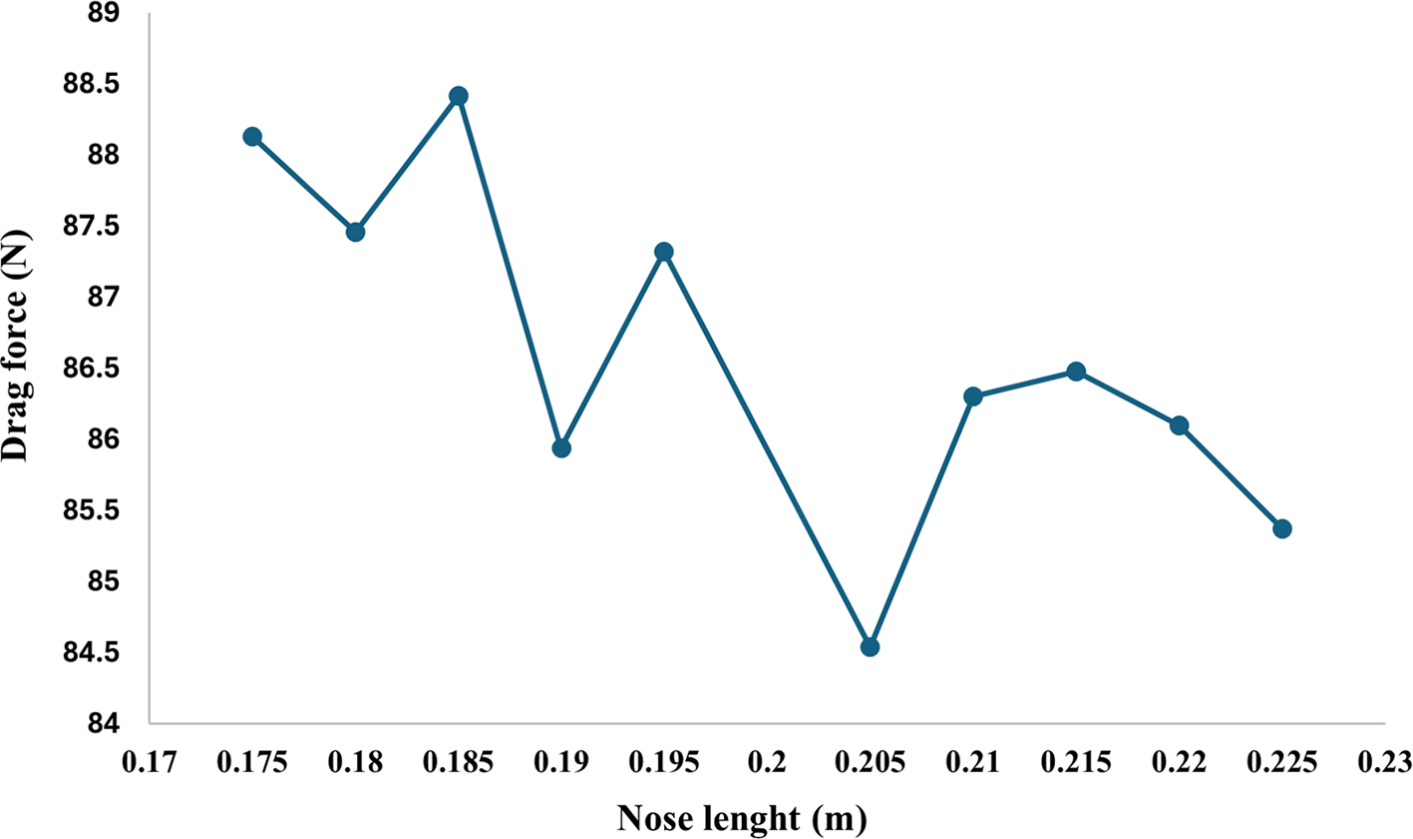
Drag force due to an increase and decrease torpedo nose length by 0.

From Figure 23 as the torpedo length increases from 0.2m the drag force will also increase at each step increment and at 0.215 m nose length the drag force reaches the maximum value of 86.48 N and after further increment the drag force gradually starts decreasing from the graph it is observed that the drag forces decrease from 86.48 N to 85.37 N at the nose length of 0.225 m. Similarly, from Figure 23 the variation in the drag force can be observed, after decreasing the nose length the drag force starts decreasing and from the graph it can be observed that the drag force reaches the minimum value 85.94 N at the nose length of 0.19m, on further decrease in length the drag starts increasing gradually.

In this simulation the thrust is not considered, here only the change in drag force due to the change in angle of attack is studied. The change in geometry always does not help because if the nose length is reduced then it is not sure that the drag force will increase. After all, the change in overall length is only very important. The length of the torpedo glider is related to total volume whereas the diameter is directly related to total volume, but the fineness ratio and diameter are not related to each other. Hence the fineness ratio can be neglected for this simulation because for that reason the nose optimization is performed, and this study mainly focuses on the design of an symmetric body which will help in reducing the drag force.

**Figure 24.**
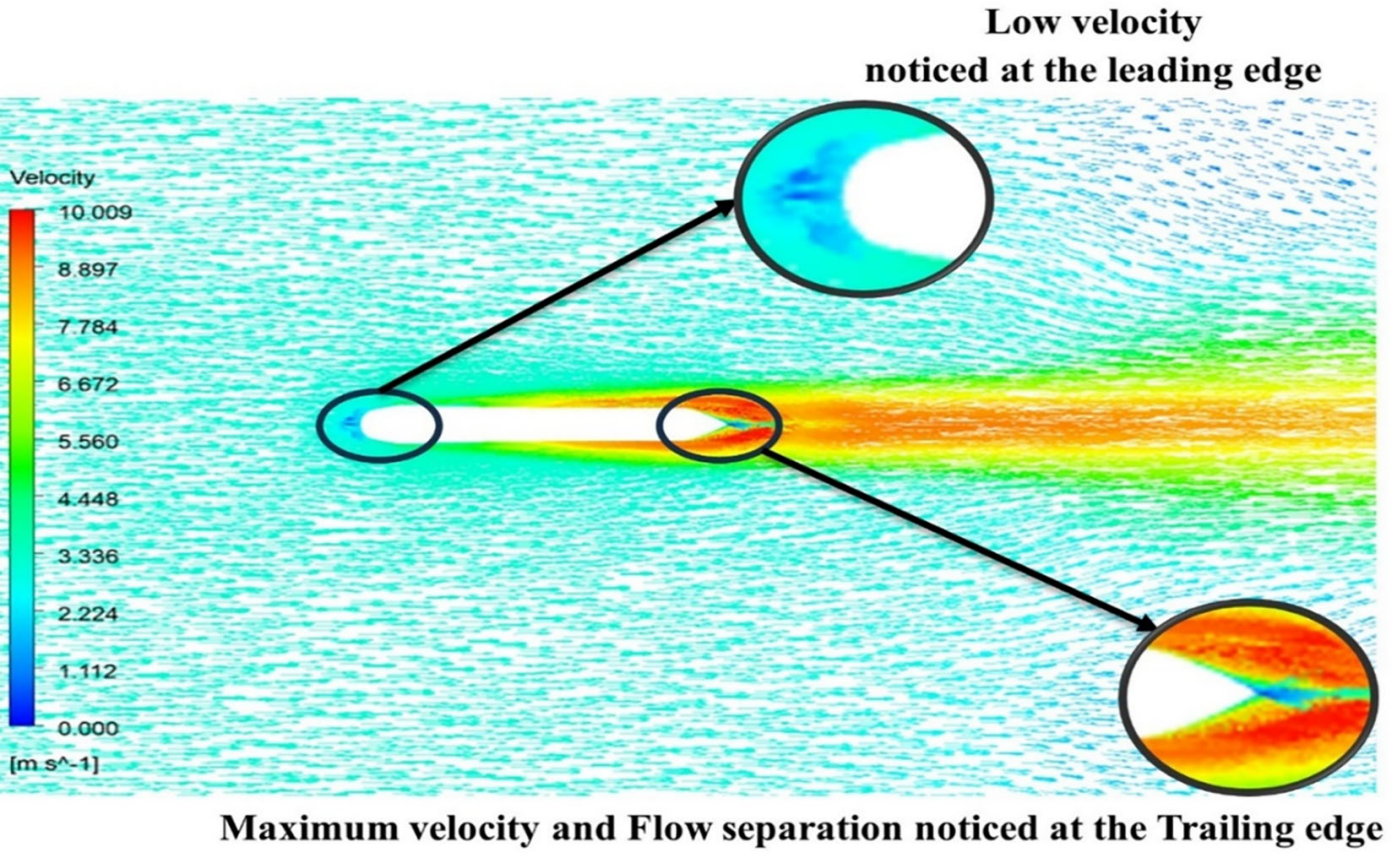
Velocity contour due to 0.205 m.

**Figure 25.**
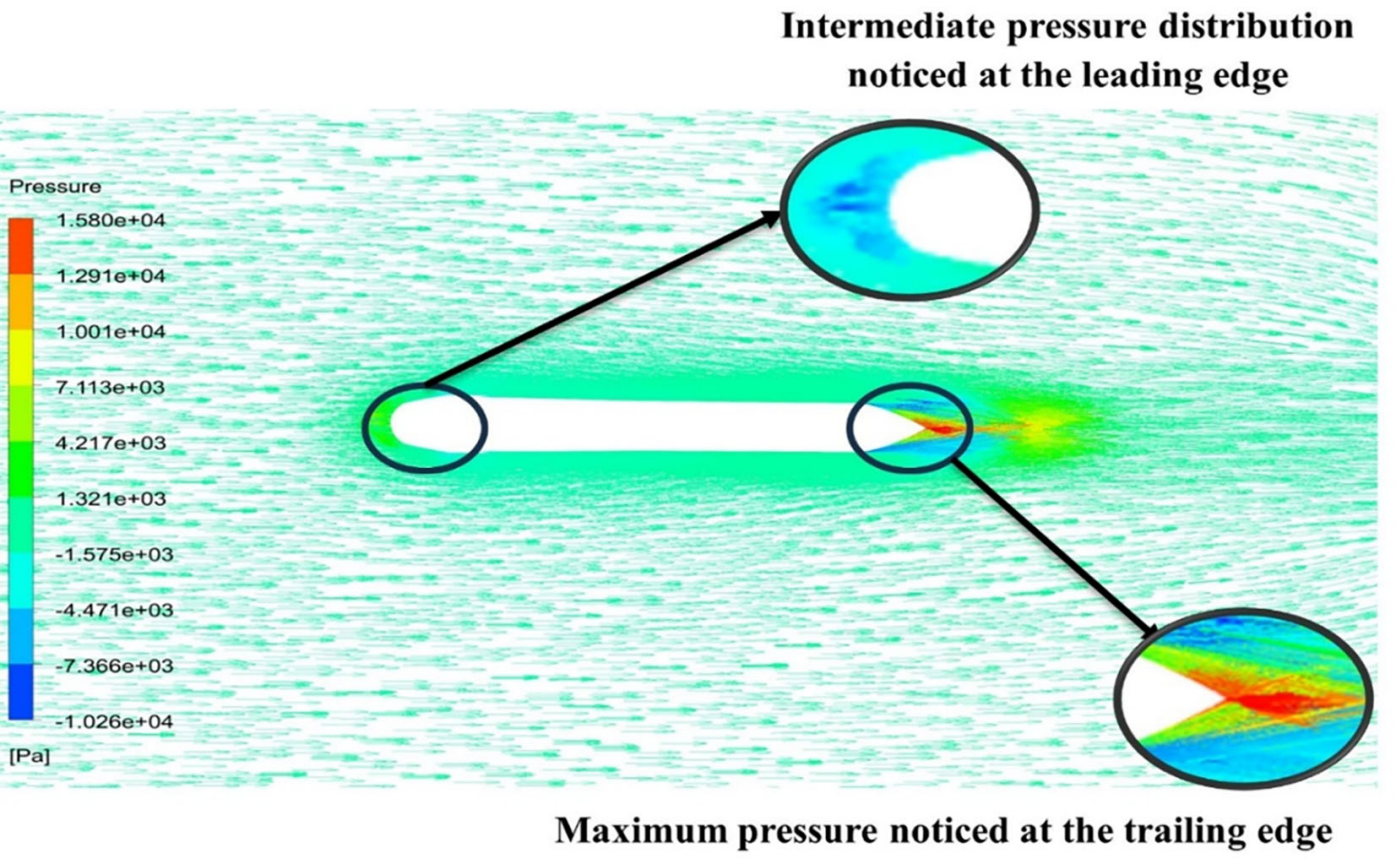
Pressure contour due to 0.205 m.

**Figure 26.**
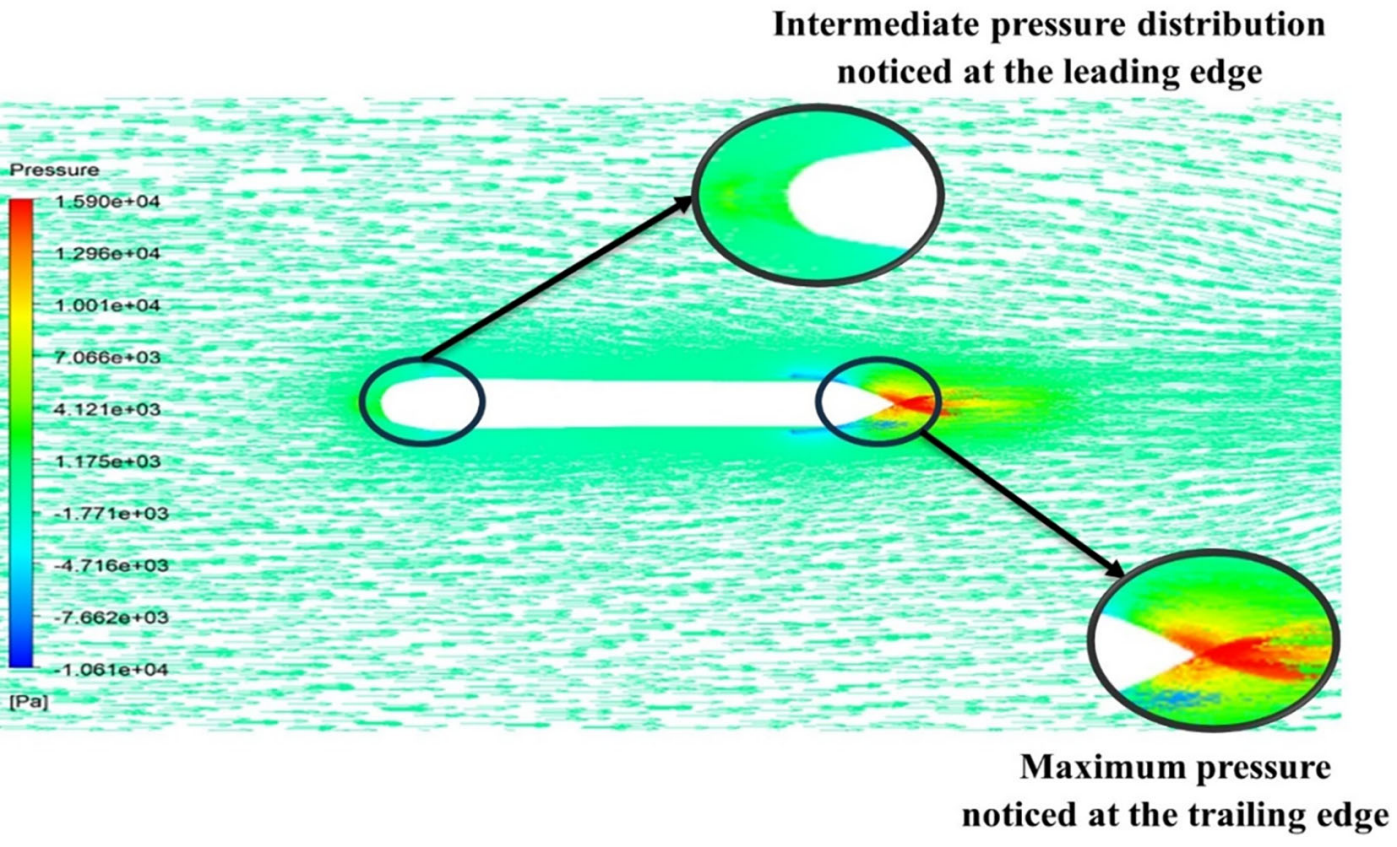
Velocity contour due to 0.19 m.

**Figure 27.**
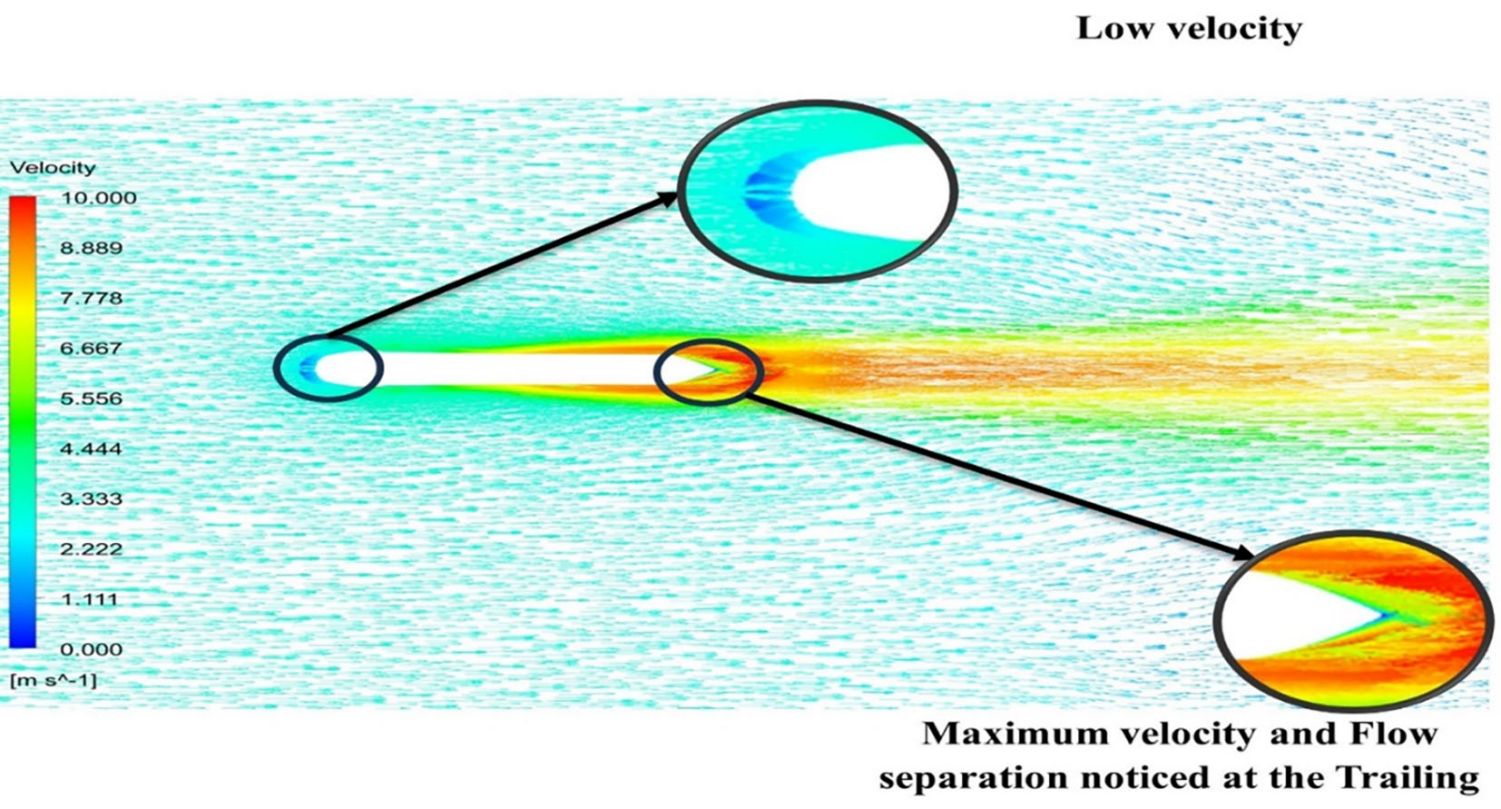
Pressure contour due to 0.19 m.

The above figure represents the velocity and pressure contours over the torpedo glider, here the same free stream velocity of 10.16 m/s is considered, and the same boundary conditions are applied. Figure 24 and Figure 25 represent the velocity and pressure contours due to a nose length of 0.205 m. Similarly, Figure 26 and Figure 27 represents the velocity and pressure contours over the torpedo glider at a velocity of 10.16 m/s for the same 0.19 m nose length. The 0.19 m and 0.205 m are chosen because it produces less drag force of 84.54 N and 87.32 N. From Figure 24 the low velocity of 2.224 m/s is observed at the leading-edge surface, maximum velocity of 10 m/s, and flow separation at the trailing edge. Similarly, an intermediate pressure between 2.398 kPa to 5.79 kPa is observed from Figure 25, and the figure also shows that the trailing edge tip has a maximum pressure of 9.818 KPa, both contour and velocity contours can be observed from Figure 24 and Figure 25. Similarly, from Figure 27 an intermediate pressure between 1.157 kPa to 10.01 kPa is observed, and the figure also shows that the trailing edge tip has a maximum pressure of 15.8 kPa, both contour and velocity contours can be observed from Figure 26 and Figure 27.

### 4.4 Results overview

**Figure 28.**
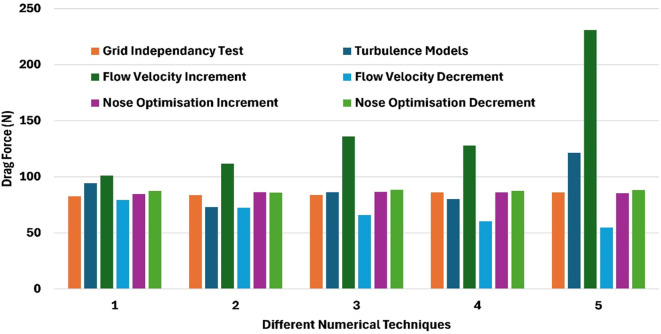
Different numerical methods with drag force.

Several numerical methods were employed and analyzed, based on Figure 28 observations. The grid independency test, conducted with a mesh consisting of million elements resulted in a drag force 86.11 N, achieving a 1.47% improvement in drag force reduction. The application of the Spallart-Allmaras model results in a drag force of 86.28 N with a 1.28% improvement in drag force reduction. Further analysis at a velocity of 7.66 m/s demonstrated an improvement in a drag force of 54.78 N, which corresponds to a 37.3% reduction. Additionally, nose optimization was performed, nose length of 0.19 m resulting in a drag force of 85.94 N with 1.67% improvement. Increasing the nose length to 0.205 m further reduced the drag force to 84.54 N, resulting in a 3.27% improvement. These observations highlight the impact of the nose optimization on drag reduction, effectiveness of velocity adjustments and selecting the suitable turbulence model in minimizing the hydrodynamic drag force.

## 5. Conclusions

In this study, the relationship between the nose geometry of the cylindrical torpedo hull and drag force is investigated using 3D CFD simulation. The study mainly focuses on drag minimization by optimizing the parabolic nose, to optimize the nose many validations are performed. The important step was selecting the proper baseline where the drag force was found to be 86.28 N. The result was validated, there was 1.47% error while comparing with the reference drag force of 87.4 N. After selecting the proper baseline, the torpedo model was simulated for different turbulence models and the Sparlart-Allmara model was chosen as the baseline model for further simulation. In the later stages, the velocities are varied to observe the variation in the drag force, the velocities are incremented from 10.16 m/s to 15.16 m/s where the maximum drag force of 230.9 N is observed. Similarly, the velocity is decreased from 10.16 m/s to 7.16 m/s where a minimum drag force of 54.78 N is observed, from the above steps it can be concluded that the drag force can be minimized by reducing the velocity, but in the defence application speed is always an important parameter. By considering the above aspects nose optimization is chosen where the torpedo glider nose is incremented from 0.2 m to 0.025 m and decrement from 0.2 m to 0.175 m. From this study it can be observed that the 0.205 m and 0.19 m nose lengths produce 84.54 N and 85.94 N drag force which is lower compared to other optimization nose lengths. While comparing with the referenced drag force the error was found to be 3.32% and 1.67% which is acceptable. From the overall simulation it can be observed that the reducing velocity does not reduce the drag, nowadays torpedo speeds are increasing and the possible hydrodynamic solution to reduce the drag force is by optimizing the tail or nose. In this study the focus was to optimize the nose because the speed was less, torpedo glider body was symmetric, and the tail was sharp with the tail angle of 20°. While optimizing the nose it is very important to consider volume and diameter, because the overall length is directly related to total volume and diameter. These design parameters also affect the drag force, hence considering this parameter for the design will help in minimizing the drag force. The future scope of this work includes conducting thermal analysis of the torpedo glider and designing the most suitable geometry. As the torpedo speeds are upgrading, thermal analysis is also essential to predict the drag. Since in the water medium the pressure is much higher compared to an air medium. Therefore, thermal analysis and optimizing the design are crucial to reduce the drag effectively.

## Ethics and consent

Ethics and consent were not required.

## Data Availability

All data underlying the results are available as part of the article and no additional source data are required.

## References

[1] Gao T Wang Y Pang Y : Hull shape optimization for autonomous underwater vehicles using CFD. *Eng. Appl. Comput. Fluid Mech.* Jan. 2016;10(1):599–607. 10.1080/19942060.2016.1224735

[2] Liu Y Ma J Ma N : Experimental and Numerical Study on Hydrodynamic Performance of an Underwater Glider. *Math. Probl. Eng.* 2018;2018:1–13. 10.1155/2018/8474389

[3] Yang L Cao J Cao J : Hydrodynamic and vertical motion analysis of an underwater glider. *OCEANS 2016 - Shanghai, China.* 2016; pp.1–6. 10.1109/OCEANSAP.2016.7485413

[4] Singh Y Bhattacharyya SK Idichandy VG : CFD approach to modelling, hydrodynamic analysis and motion characteristics of a laboratory underwater glider with experimental results. *J. Ocean Eng. Sci.* Jun. 2017;2(2):90–119. 10.1016/j.joes.2017.03.003

[5] Singh Y Bhattacharyya SK Idichandy VG : CFD approach to steady state analysis of an underwater glider. *2014 Oceans-St. John’s.* IEEE;2014.

[6] Zhang Y Zhang Z Quan Z Liu GL : Hydrodynamic performance and calculation of lift–drag ratio on underwater glider. *J. Mar. Sci. Technol.* Mar. 2021;26(1):16–23. 10.1007/s00773-020-00716-7

[7] Stryczniewicz K Drężek P : CFD Approach to Modelling Hydrodynamic Characteristics of Underwater Glider. *Trans. Aerosp. Res.* Dec. 2019;2019(4):32–45. 10.2478/tar-2019-0021

[8] Cai X Wu W Han W : Study on Water Entry of a 3D Torpedo Based on the Improved Smoothed Particle Hydrodynamics Method. *Appl. Sci.* Jun. 2024;14(11). 10.3390/app14114441

[9] Keerthi Raaj S Saha N Sundaravadivelu R : Exploration of deep-water torpedo anchors - A review. *Ocean Eng.* Elsevier Ltd;Feb. 15, 2023;270. 10.1016/j.oceaneng.2022.113607

[10] Gartner N Richier M Dune C : Hydrodynamic Parameters Estimation Using Varying Forces and Numerical Integration Fitting Method. *IEEE Robot Autom. Lett.* 2022;7:11713–11719. 10.1109/LRA.2022.3205126

[11] Özgören M : Analysis of Attack Angle Effect on Flow Characteristics Around Torpedo-Like Geometry Placed Near the Free-Surface via CFD. *Politeknik Dergisi.* Dec. 2021;24(4):1579–1592. 10.2339/politeknik.675632

[12] Le TL Hong DT : Computational fluid dynamics study of the hydrodynamic characteristics of a torpedo-shaped underwater glider. *Fluids.* Jul. 2021;6(7). 10.3390/fluids6070252

[13] Divsalar K Shafaghat R Farhadi M : Experimental analysis on hydrodynamic coefficients of an underwater glider with spherical nose for dynamic modeling and motion simulation. *SN Appl Sci.* Feb. 2021;3(2). 10.1007/s42452-021-04241-z

[14] Kenan O Yanıktepe B Sekeroglu E : POD Analysis of Flow around Torpedo Like Geometry with a Hemispherical Nose. *Osmaniye Korkut Ata Üniversitesi Fen Bilimleri Enstitüsü Dergisi.* 2023;6(Ek Sayı):555–566. 10.47495/okufbed.1371217

[15] Shashank Shankar RV Vijayakumar R : CFD estimation of HDCs for varying bodies of revolution of underwater gliders. *Ocean Syst. Eng.* 2023;13(3):269–286. 10.12989/ose.2023.13.3.269

[16] Sarigiguzel F : Experimental investigation of free-surface effects on flow characteristics of a torpedo-like geometry having a cambered nose. *Ocean Eng.* Jun. 2022;253:111174. 10.1016/j.oceaneng.2022.111174

[17] Zhang B Fu Y Zhang MH : Numerical investigations into the influence of geometric configurations on hydrodynamic characteristics of torpedo anchors. *Mar. Struct.* Jul. 2023;90:103445. 10.1016/j.marstruc.2023.103445

[18] Won DJ Kim J Kim J : Design optimization of duct-type AUVs using CFD analysis. *Intell. Serv. Robot.* Oct. 2015;8(4):233–245. 10.1007/s11370-015-0179-9

[19] Soh BP Pao W Al-Kayiem HH : Numerical analyses for improved hydrodynamics of deep-water torpedo anchor. *IOP Conference Series: Materials Science and Engineering.* Institute of Physics Publishing;Dec. 2015. 10.1088/1757-899X/100/1/012059

[20] Choi JH Penmetsa RC Grandhi RV : Shape optimization of the cavitator for a supercavitating torpedo. *Struct. Multidiscip. Optim.* Feb. 2005;29(2):159–167. 10.1007/s00158-004-0466-0

[21] Cao L Zhu J Zeng G : Viscous-flow calculations of submarine maneuvering hydrodynamic coefficients and flow field based on same grid topology. *J. Appl. Fluid Mech.* 2016;9(2):817–826. 10.18869/acadpub.jafm.68.225.24570

[22] Yang M Wang Y Yang S : Shape optimization of underwater glider based on approximate model technology. *Appl. Ocean Res.* 2021;110: 102580. 10.1016/j.apor.2021.102580

[23] Yang M Wang Y Zhang X : Parameterized Dynamic Modeling and Spiral Motion Pattern Analysis for Underwater Gliders. *IEEE J. Oceanic Eng.* 2023;48(1):112–126. 10.1109/JOE.2022.3181896

[24] Sun T Chen G Yang S : Design and optimization of a bio-inspired hull shape for AUV by surrogate model technology. *Eng. Appl. Comput. Fluid Mech.* 2021;15(1):1057–1074. 10.1080/19942060.2021.1940287

[25] Wang Y Wang C Yang M : Glide performance analysis of underwater glider with sweep wings inspired by swift. *Front. Mar. Sci.* 2022;9. 10.3389/fmars.2022.1048328

[26] Liu M Song Y Yang S : Rapid data-driven individualised shape design of AUVs based on CFD and machine learning. *Ships Offshore Struct.* 2024;19(12):2050–2064. 10.1080/17445302.2024.2323884

[27] Şerifoğlu MO Tutak B : Drag force-internal volume relationship for underwater gliders and drag coefficient estimation using machine learning. *Ocean Eng.* Oct. 2022;262:112325. 10.1016/j.oceaneng.2022.112325

[28] Ting MC Abdul Mujeebu M Abdullah MZ : Numerical Study on Hydrodynamic Performance of Shallow Underwater Glider Platform. 2012.

