# Supplementary material for: Hydrodynamic Performance Enhancement of Torpedo-Shaped Underwater Gliders Using Numerical Techniques
Source: F1000Res. 2025 Apr 23;13:1274. Originally published 2024 Oct 24. [Version 2] doi: 10.12688/f1000research.154040.2 (PMC12163899; doi:10.12688/f1000research.154040.2)
Supplement: Peer review history

# Reviewer report: Reviewer response for version 2

**DOI:** 10.5256/f1000research.179622.r380916

**Published:** 2025-05-15

**Authors:** Ajith Raj Rajendran

**Affiliations:** 1 Department of Aerospace Engineering, Karunya University, Coimbatore, Tamil Nadu, India

The manuscript presents a detailed numerical investigation into the hydrodynamic performance of a torpedo-shaped underwater glider. A symmetric glider model was developed and analyzed using the licensed ANSYS Fluent 20.1 software. The study includes a series of simulations conducted under various flow conditions, incorporating a grid independence study, the evaluation of multiple turbulence models, and systematic variations in inflow boundary conditions. Specifically, inflow velocity was varied from 10.16 m/s to 15.16 m/s in 1 m/s increments and from 10.16 m/s to 7.66 m/s in 0.5 m/s decrements. A total of six turbulence models were assessed.

 Among the turbulence models analyzed, the Spalart–Allmaras model exhibited the highest level of accuracy, producing a minimum validation error of 1.28%. A notable aspect of the research is the investigation into nose geometry optimization for drag reduction. Two nose lengths—0.205 m and 0.19 m—were evaluated, resulting in validation errors of 2.81% and 1.16%, respectively. These results are clearly presented and thoroughly discussed in the subsequent sections of the manuscript.

 The study effectively examines the impact of various design modifications on drag force reduction. The use of the Spalart–Allmaras model significantly improved accuracy, with a validation error of 1.28%. Moreover, reducing the inflow velocity led to a substantial decrease in drag, with a maximum improvement of 37.3%. Nose geometry optimization further enhanced hydrodynamic performance, with the 0.205 m nose yielding a 3.37% drag reduction, and the 0.19 m nose producing a 1.67% reduction. These findings offer valuable insights for researchers involved in underwater vehicle design and hydrodynamic optimization.

Is the work clearly and accurately presented and does it cite the current literature?

Yes

If applicable, is the statistical analysis and its interpretation appropriate?

Yes

Are all the source data underlying the results available to ensure full reproducibility?

Yes

Is the study design appropriate and is the work technically sound?

Yes

Are the conclusions drawn adequately supported by the results?

Yes

Are sufficient details of methods and analysis provided to allow replication by others?

Yes

Reviewer Expertise:

Material Science, Aerodynamics

I confirm that I have read this submission and believe that I have an appropriate level of expertise to confirm that it is of an acceptable scientific standard.

# Reviewer report: Reviewer response for version 2

**DOI:** 10.5256/f1000research.179622.r380915

**Published:** 2025-05-03

**Authors:** Hari Warrior

**Affiliations:** 1 Department of Ocean Engineering and Naval Architecture, Indian Institute of Technology Kharagpur, Kharagpur, West Bengal, India

This is a good paper on the applications of nose optimization on hydrodynamic performance. The work is acceptable if some questions are addressed.

 1) Kindly split the drag into a pressure drag and viscous drag and study how these individual components change. This would explain the variation in total drag, especially when it might increase and then decrease.

 2) I think equations 1 to 5 require a workover.  Please check the indices and please mention what v stands for. Is it velocity or eddy viscosity?

 Please make the corrections and resubmit it.

Is the work clearly and accurately presented and does it cite the current literature?

Partly

If applicable, is the statistical analysis and its interpretation appropriate?

Not applicable

Are all the source data underlying the results available to ensure full reproducibility?

Partly

Is the study design appropriate and is the work technically sound?

Yes

Are the conclusions drawn adequately supported by the results?

Partly

Are sufficient details of methods and analysis provided to allow replication by others?

Yes

Reviewer Expertise:

Ocean Engineering, Computational Fluid Dynamics

I confirm that I have read this submission and believe that I have an appropriate level of expertise to confirm that it is of an acceptable scientific standard, however I have significant reservations, as outlined above.

# Reviewer report: Reviewer response for version 2

**DOI:** 10.5256/f1000research.179622.r380913

**Published:** 2025-04-30

**Authors:** Sandeep Juluru

**Affiliations:** 1 Department of Aeronautical Engineering, Sandip University, Nashik, Maharashtra, India

The authors conducted a study on the Hydrodynamic Performance Enhancement of Torpedo-Shaped Underwater Gliders Using Numerical Techniques. This research employed various numerical methods to investigate the hydrodynamic characteristics of a symmetric torpedo-shaped glider. The glider model was developed and analyzed using a licensed version of the ANSYS Fluent 20.1 software.

 The study explored the glider’s behavior under different flow conditions, including grid sensitivity tests, variations in turbulence models, and changes in inflow boundary conditions. Specifically, the inflow velocity was varied from 10.16 m/s to 15.16 m/s in 1 m/s increments, and from 10.16 m/s to 7.66 m/s in 0.5 m/s decrements. Additionally, six different glider models were examined.

 The research also involved testing various turbulence models, with the Spalart-Allmaras model yielding the lowest validation error of 1.28%, focusing particularly on nose optimization. To minimize drag, the nose length was varied, with lengths of 0.205 m and 0.19 m producing validation errors of 2.81% and 1.16%, respectively. Detailed results and analyses are presented in the subsequent sections of this article.

 In conclusion, this study comprehensively evaluated several modifications and their impacts on drag force reduction. The novelty of the work is maintained through the application of different numerical techniques, and all results are validated and presented with a high level of detail and realism.

Is the work clearly and accurately presented and does it cite the current literature?

Yes

If applicable, is the statistical analysis and its interpretation appropriate?

Partly

Are all the source data underlying the results available to ensure full reproducibility?

Yes

Is the study design appropriate and is the work technically sound?

Yes

Are the conclusions drawn adequately supported by the results?

Yes

Are sufficient details of methods and analysis provided to allow replication by others?

Yes

Reviewer Expertise:

My area of research is in Aerodynamic using CFD

I confirm that I have read this submission and believe that I have an appropriate level of expertise to confirm that it is of an acceptable scientific standard.

# Reviewer report: Reviewer response for version 1

**DOI:** 10.5256/f1000research.169017.r337534

**Published:** 2024-11-23

**Authors:** Denghui Qin

**Affiliations:** 1 Northwestern Polytechnical University, Xi’an, China

This article presents a numerical simulation study on the influence of different head shapes on the resistance of torpedo gliders. Among them, the results of different turbulence models and different head lengths were compared.

 Comments about this paper:

 1. Figures 2 have no meaning. A scientific paper does not need to introduce literature reading and identify problems in this way.

 2. Figures 3: If the author has basic knowledge of fluid mechanics, they should be aware that the resistance of the torpedo model shown in Figure 3 will be significant, as its nose does not even have a streamlined transition with its body. It is unreasonable to use a curve with high resistance as a baseline.

 3. Figure 5: Why are the models of the two baselines different?

 4. Table 1: These are common knowledge and do not need to be listed separately

 5. Figure 8 should provide a detailed description of the size of the computational domain, as it is crucial for CFD calculations. Figures 8, 9, and 10 can all be combined into one image.

 6. Figure 11 shows that the number of grids for grid independence verification should be based on the magnification factor, rather than 4.1-4.5 million

 7. Figure 14-28, those figures are not suitable CFD Contours. Please refer to more CFD papers for graphics.

 Based on the comments, this paper is not proper to be indexed.

Is the work clearly and accurately presented and does it cite the current literature?

Partly

If applicable, is the statistical analysis and its interpretation appropriate?

No

Are all the source data underlying the results available to ensure full reproducibility?

Partly

Is the study design appropriate and is the work technically sound?

Partly

Are the conclusions drawn adequately supported by the results?

Partly

Are sufficient details of methods and analysis provided to allow replication by others?

Yes

Reviewer Expertise:

Fluid Mechanics

I confirm that I have read this submission and believe that I have an appropriate level of expertise to state that I do not consider it to be of an acceptable scientific standard, for reasons outlined above.

# Reviewer report: Reviewer response for version 1

**DOI:** 10.5256/f1000research.169017.r337532

**Published:** 2024-11-22

**Authors:** Shaoqiong Yang

**Affiliations:** 1 Tianjin University, Tianjin, China

***Review comments on the manuscript (Hydrodynamic Performance Enhancement of Torpedo-Shaped Underwater Gliders Using Numerical Techniques)***

 In the manuscript (#Hydrodynamic Performance Enhancement of Torpedo-Shaped Underwater Gliders Using Numerical Techniques), the drag reduction effect resulting from the shape optimization of the nose of a torpedo-shaped underwater vehicle was investigated via the numerical simulation method of CFD to minimize the drag of the nose. Meanwhile, based on the optimization results, the key techniques for effectively reducing the drag of underwater vehicles were identified. This manuscript provides methodological support for the hull shape design of a torpedo-shaped underwater vehicle using CFD methods, and the results of the study also have significant implications for future research on hull shape optimization. However, there are some problems with the description in the manuscript. If the problems in the manuscript can be explained and corrected in the revised version, it can be indexed.

 1. In the manuscript, the authors used the term “aerodynamic performance” several times, but for the study of underwater vehicles, it is recommended to use “hydrodynamic performance” for description.

 2. The figures presented in the manuscript (e.g., Figure 3) have relatively low resolution. It is suggested that figures with higher resolution be employed for display. 

 3. In section 3.1, it is advised to provide more detailed information on the optimization method used.

 4. In section 3.1.4, it is suggested that the authors introduce the setting process of the working condition parameters in greater detail. 

 5. In Figure 12, the curve used to represent the " *k*-elipson Standard model" is not shown in the figure, and it is recommended to make corrections. At the same time, 'elipson' should be 'epsilon'; it is suggested to re-examine.

 6. The results in Figures 23 and 24 are suggested to be combined into one figure to observe more comprehensively the influence of the changes in the bow length on the drag.  

 7. The speed of underwater gliders is generally relatively low, can it reach over 10m/s?

 8. By the way, we give some papers about the shape optimization based on CFD to authors for reference this time or communications in future:[1],[2],[3],[4],[5]

Is the work clearly and accurately presented and does it cite the current literature?

Partly

If applicable, is the statistical analysis and its interpretation appropriate?

Yes

Are all the source data underlying the results available to ensure full reproducibility?

No source data required

Is the study design appropriate and is the work technically sound?

Yes

Are the conclusions drawn adequately supported by the results?

Yes

Are sufficient details of methods and analysis provided to allow replication by others?

Partly

Reviewer Expertise:

My studies mainly focus on design theory, novel drag-reduction technology, and sea trial & marine application of unmanned underwater vehicles, especially underwater gliders.

I confirm that I have read this submission and believe that I have an appropriate level of expertise to confirm that it is of an acceptable scientific standard, however I have significant reservations, as outlined above.

## References

[1] : Shape optimization of underwater glider based on approximate model technology. *Applied Ocean Research* .2021;110: 10.1016/j.apor.2021.102580 10.1016/j.apor.2021.102580

[2] : Parameterized Dynamic Modeling and Spiral Motion Pattern Analysis for Underwater Gliders. *IEEE Journal of Oceanic Engineering* .2023;48(1) : 10.1109/JOE.2022.3181896 112-126 10.1109/JOE.2022.3181896

[3] : Design and optimization of a bio-inspired hull shape for AUV by surrogate model technology. *Engineering Applications of Computational Fluid Mechanics* .2021;15(1) : 10.1080/19942060.2021.1940287 1057-1074 10.1080/19942060.2021.1940287

[4] : Glide performance analysis of underwater glider with sweep wings inspired by swift. *Frontiers in Marine Science* .2022;9: 10.3389/fmars.2022.1048328 10.3389/fmars.2022.1048328

[5] : Rapid data-driven individualised shape design of AUVs based on CFD and machine learning. *Ships and Offshore Structures* .2024; 10.1080/17445302.2024.2323884 1-15 10.1080/17445302.2024.2323884

# Response

**Published:** 2025-03-21

**Authors:** SRINIVAS G

**Affiliations:** DEPARTMENT OF AERO & AUTO ENGG, Manipal Academy of Higher Education, Manipal, Karnataka, India

1.In the manuscript, the authors used the term “aerodynamic performance” several times, but for the study of underwater vehicles, it is recommended to use “hydrodynamic performance” for description **.**

**Response: As per your advice, the changes have been implemented throughout the manuscript.**

 2.The figures presented in the manuscript (e.g., Figure 3) have relatively low resolution. It    is suggested that figures with higher resolution be employed for display. 

**Response: The high-resolution image has been replaced and updated in the manuscript.**

 Please refer the image here - https://f1000research.s3.amazonaws.com/linked/717197.154040-Image_1.pdf 

 3. In section 3.1, it is advised to provide more detailed information on the optimization method used.

**Response: The optimization of the nose shape design has been explained in detail and updated in the new version.**

 4. In section 3.1.4, it is suggested that the authors introduce the setting process of the working condition parameters in greater detail. 

**Response: The** **following sentences have been added to the new version of the article under Section 3.1.4:**

 "The vorticity-based Spalart-Allmaras model was chosen for the analysis, with aluminum as the glider material. The inlet was defined as a velocity inlet with a turbulence intensity of 10% and a hydraulic diameter of 2 m. The outlet was specified as a pressure outlet with a backflow turbulence ratio of 10. Both the glider and walls were treated as fixed walls with a roughness constant of 0.5. The SIMPLE algorithm was selected as the solution method for the baseline analysis."

 5. In Figure 12, the curve used to represent the " *k*-elipson Standard model" is not shown in the figure, and it is recommended to make corrections. At the same time, 'elipson' should be 'epsilon'; it is suggested to re-examine.

**Response: The corrections have been made as per the suggestions and incorporated into the new version of the article. Additionally, a new graph has been plotted using MATLAB.**

 Please refer to the new graph here - https://f1000research.s3.amazonaws.com/linked/717198.154040_-_New_Graph.pdf

 6. The results in Figures 23 and 24 are suggested to be combined into one figure to observe more comprehensively the influence of the changes in the bow length on the drag.

**Response: The velocity increment and decrement graphs have been combined into a single graph, and the updated graph has been included in the research paper.**

 Please refer to the updated graph here - https://f1000research.s3.amazonaws.com/linked/717199.154040_-_Updated_Graph.pdf

 7. The speed of underwater gliders is generally relatively low, can it reach over 10m/s?

**Response: it is possible. As per the reference paper, the free stream velocity was mentioned to be possible up to 14 m/s. This article focuses on varying the velocity from 11.16 m/s to 15.16 m/s, as well as decreasing the velocity from 9.66 m/s to 7.66 m/s to evaluate performance variations.**

 8.By the way, we give some papers about the shape optimization based on CFD to authors for reference this time or communications in future:[1],[2],[3],[4],[5]

**Responses: The suggested research papers have been cited in the present study.**
